# Plant palatability and trait responses to experimental warming

**DOI:** 10.1038/s41598-020-67437-0

**Published:** 2020-06-29

**Authors:** Tomáš Dostálek, Maan Bahadur Rokaya, Zuzana Münzbergová

**Affiliations:** 1 Institute of Botany, The Czech Academy of Sciences, Zámek 1, 252 43 Průhonice, Czech Republic; 2 Department of Botany, Faculty of Science, Charles University, Benátská 2, 128 01 Prague, Czech Republic; 3 Department of Biodiversity Research, Global Change Research Centre, The Czech Academy of Sciences, Bělidla 4a, 603 00 Brno, Czech Republic

**Keywords:** Plant ecology, Climate-change ecology

## Abstract

Climate warming is expected to significantly affect plant–herbivore interactions. Even though direct effects of temperature on herbivores were extensively studied, indirect effects of temperature (acting via changes in host plant quality) on herbivore performance have rarely been addressed. We conducted multiple-choice feeding experiments with generalist herbivore *Schistocerca gregaria* feeding on six species of genus *Impatiens* cultivated at three different temperatures in growth chambers and a common garden. We also studied changes in leaf morphology and chemistry. We tested effects of temperature on plant palatability and assessed whether the effects could be explained by changes in the leaf traits. The leaves of most *Impatiens* species experienced the highest herbivory when cultivated at the warmest temperature. Traits related to leaf morphology (specific leaf area, leaf dry matter content and leaf area), but not to leaf chemistry, partly mediated the effects of temperature on plant palatability. Herbivores preferred smaller leaves with lower specific leaf area and higher leaf dry matter content. Our study suggests that elevated temperature will lead to changes in leaf traits and increase their palatability. This might further enhance the levels of herbivory under the increased herbivore pressure, which is forecasted as a consequence of climate warming.

## Introduction

Insect herbivores have significant impacts on performance of plant populations^1,2^. The strength of this interaction may be modified as a consequence of global climate change. In high altitudes, the climate change is expected to cause increase in temperature^3^ with many direct effects on insects^4–6^. Higher temperatures are predicted to increase population densities of most insect species and cause alterations in their body size, life cycle duration and in the extent of their host plant exploitation^7^ strengthening the intensity of the plant–herbivore interactions (e.g.,^7,8^). However, other studies showed that warming can reduce plant–herbivore interactions in some systems, and the responses thus seem to be species specific^5,6,9,10^. A possible explanation for this specificity is the differential effects of temperature on plant traits that determine plant palatability. This issue is, however, still largely unexplored (but see Descombes et al.^9^).

The effects of temperature on plant palatability are often studied along altitudinal gradients^11^. Plants along these natural temperature gradients experience different levels of herbivory, leading to changes in the levels of their defences^12,13^. For example, plants from warmer conditions produce leaves that are harder to consume (thicker, or with higher trichome density^14^), have lower nitrogen and phosphorus contents^15^ or contain larger amounts of secondary metabolites^12^. However, most of the above-described studies are from natural environments where other factors except for temperature vary as well (such as intensity of UVB radiation, precipitation or abundance of herbivores). The differences in plant traits among these environments may thus also be caused by these other factors. To what extent it is truly the temperature that is responsible for the observed trait differentiation is thus often unknown (but see^9,16,17^). In addition, field studies alone do not allow to distinguish whether the trait differentiation is due to phenotypic plasticity or genetic differentiation among the plants^18^. It is thus not clear whether the trait differentiation among plants is due to the response of the plants to their actual environment or whether it is, for example, due to selection by some past environmental conditions (but see, e.g., ^11,19^).

Previous studies have often found contrasting results when comparing the effects of environment on plant–herbivore interactions among different plant species, indicating that plant species and environmental conditions interact to determine plant palatability^6,9,20,21^. For example, Lemoine et al.^6^ showed that overall consumption rates increased with temperature in comparison of wide range of herbivore-plant pairs. However, there was substantial variation in thermal responses among individual pairs. Descombes et al.^9^ found that warming modified physical and chemical phenotypes of all studied plant species. However, the trait changes did not result in a consistent effect on plant resistance against herbivores. Further studies comparing the relationships between temperature and plant palatability among multiple species are thus needed to assess the generality of the temperature effects. By simultaneously exploring the differences in plant traits, it should be possible to identify the mechanisms underlying the temperature effects.

In this study, we explored the effects of temperature on palatability and traits [specific leaf area (SLA), leaf dry matter content (LDMC), leaf area, content of carbon (C), nitrogen (N) and phosphorus (P) in leaf biomass] of six closely related plant species of the genus *Impatiens,* Balsaminaceae, that grow naturally in the Himalayas. Using a generalist omnivore desert locust (*Schistocerca gregaria*) as an herbivore^22^, we conducted multiple-choice feeding experiments with the plants cultivated at three different temperatures in growth chambers (representing temperatures experienced by the six *Impatiens* species along their natural altitudinal range and the expected climate warming) and in a common garden (representing more natural conditions). We then assessed the effects of temperature on plant palatability and plant traits. In particular, we asked the following questions: (1) What is the effect of temperature of plant cultivation on plant traits and plant palatability? (2) Can the differences in plant palatability be explained by the differences in plant traits? (3) What are the differences in plant traits and palatability among the *Impatiens* species, and do the effects of plant species identity interact with the effects of the temperature of plant cultivation?

We hypothesized that the temperature of plant cultivation will affect morphological (such as SLA, LDMC and leaf area) and chemical (such as content of C, N and P) traits of plant leaves. These changes will lead to changes in plant palatability among the plants cultivated under different conditions. Since each of the six species used as model naturally grows under different environmental conditions, we assume that *Impatiens* species will differ in their responses to temperature. However, the general relationship between plant traits and plant palatability will be the same.

## Material and methods

### Seed sampling

We used six species of the genus *Impatiens*, Balsaminaceae family: *I. balsamina* L., *I. racemosa* DC., *I. scullyi* Hook.f., *I. tricornis* Lindl. (before revision by Akiyama et Ohba^23^, usually called *I. scabrida*), *I. falcifer* Hook.f., and *I. devendrae* Pusalkar (Supplementary Table S1). All the species are annuals native to Himalayas (Nepal, India). We selected this plant group because it is a species-rich group with many species co-occurring in similar habitats along wide altitudinal ranges with a strong temperature gradient^24^. In addition, the interactions of several species from the genus *Impatiens* with herbivores have been previously intensively studied^25,26^. For example, Gruntman et al.^25^ recorded resistance to the generalist herbivore coupled with production of specific secondary defence compounds at *I. glandulifera*. Herbivory was also suggested to play an important role (together with frost resistance) in shaping the distributional patterns of several *Impatiens* species along altitudinal gradients^27,28^. The genus *Impatiens* also contains several invasive species^29,30^ that have strong effects on native diversity^31,32^. Therefore, there is a need for novel insights into the drivers of plant–herbivore interactions within this group.

Seeds of the six *Impatiens* species were collected from natural populations in Nepalese Himalayas in autumn 2017. Seeds of each species were collected from at least five plants (twenty seeds per plant) from one population consisting of at least several tens of individuals (see Supplementary Table S1 for details). The species were selected from a larger species collection from the region. We selected species differing in their altitudinal distribution in order to present species with different temperature niches since there is close negative correlation between altitude and temperature in high altitudes in the Himalayas^12^ and thus, possibly, different plant–herbivore interactions^9,33^. The selection of species was partly limited by ability of the plants to survive in all our experimental conditions (see below) and produce sufficient number of leaves to be used in the experiments.

### Plant cultivation

*Impatiens* seeds were stratified on wet filter paper in a refrigerator (4 °C) until germination^34^. Sets of three germinated seeds of the same species were transplanted into 5 × 5 × 8.5 cm pots filled with a mixture of common garden soil and sand (1:2). For each species, we planted seeds into 15 pots in total, and the pots were distributed among three growth chambers (Vötsch Bioline 1014, Weiss Umwelttechnik GmbH, Reiskirchen, Germany) with different temperature regimes (5 pots per species and growth chamber). Although we tried to place seeds from the same mother plants in the different growth chambers, this was not always possible due to low seed germination rates. After two weeks, the seedlings were thinned to one seedling per pot. As a result, there were five individuals of each of the six *Impatiens* species in each of the three growth chambers, i.e., 90 individuals in total.

The temperature regimes in the growth chambers were set to represent present and future temperatures at localities where *Impatiens* species naturally grow in their native range in Nepal. The temperature regimes in the growth chambers were as follows: (1) cold regime (mean, minimum and maximum temperatures of 12, 6 and 17.5 °C) corresponding to temperatures from March to June at 2,700 m a. s. l., representing the median of the higher altitudinal range of *Impatiens* species in Nepal, (2) warm regime (mean, minimum and maximum temperatures of 18, 12 and 22.5 °C) corresponding to temperatures from March to June at 1,800 m a. s. l., representing the median of the lower altitudinal range of *Impatiens* species in Nepal, and (3) warm2050 regime (mean, minimum and maximum temperatures of 21, 15 and 25 °C) corresponding to temperatures from March to June at 1,800 m a. s. l., representing the median of the lower altitudinal range of *Impatiens* species in Nepal in 2050 as predicted by the global climate model MIRO5C under the greenhouse gas concentration trajectory RCP8.5^35^. This model has been used in other studies in Himalayas^36^ and represents maximum predicted temperature increase. A list of *Impatiens* species and information on their altitudinal ranges in Nepal was obtained from the Annotated Checklist of the Flowering Plants of Nepal (https://www.efloras.org/flora_page.aspx?flora_id=110), which is an updated online version of Press et al.^24^. Monthly data on the mean, minimum and maximum temperatures were obtained from the WorldClim database^37^. Data on mean temperatures at particular altitudes (medians of higher and lower altitudinal ranges of *Impatiens* species in Nepal) were obtained from the slopes of the correlations between altitudes and mean temperatures for 35 data points generated 100 m of altitudinal apart along each of four valleys in central and eastern Nepal where our seed collections took place. We used mean temperatures from March to June since this represents the premonsoon period when most *Impatiens* species germinate and start to grow. For all the temperature regimes during the whole experiment, a daily temperature course was simulated (Supplementary Fig. S1), and the same day length and radiation were used, i.e., 12 h of light (06.00–18.00 h; 250 μmol m^-2^ s^-1^) and 10 h of full dark with a gradual change in light availability in the transition between the light and dark period over 1 h. The pots were regularly watered with tap water.

We also cultivated the same plant species in a common garden of the Institute of Botany, Czech Academy of Sciences, in Průhonice, Czech Republic (49°59ʹ38ʺN, 14°33ʹ57ʺE). The garden is located 320 m above sea level in temperate climate zone, with a mean annual temperature of 8.6 °C and precipitation of 610 mm. The mean, minimum and maximum temperatures in June–August in the garden (3 months before leaf collection) were 20.7, 12.3 and 29.9 °C, respectively. Seeds in the common garden environment were sown into 5 L pots with the same soil as used in the growth chambers in January 2018, and thus, they were naturally stratified. In April, the seedlings were thinned to one seedling per pot. There were five plants of each of the six *Impatiens* species, i.e., 30 plants in total. The pots were regularly watered. The plants were protected from natural herbivory using nylon cages.

### Palatability experiments

Because the palatability was studied using only plant leaves, we will talk about leaf palatability in the subsequent text. In line with this all our traits will be measured on leaves, and we will thus talk about leaf traits below.

We performed two experiments within our study, both comparing the leaf palatability (recorded as proportion of leaf area eaten by desert locust) in common arenas (for details see below). In the first experiment (Experiment 1), we tested how leaf palatability is affected by the leaf traits of plants cultivated in growth chambers under different temperatures. In the second experiment (Experiment 2), we primarily focused on comparing leaf palatability among the six *Impatiens* species cultivated under different regimes. Both experiments were set up to study the effects of plant species identity as well as the temperature of plant cultivation and their interaction on leaf palatability. The first experiment, however, places more emphasis on comparing the temperature regimes, while the second places more emphasis on between-species comparisons. While we initially intended to perform both experiments within one larger experiment, this was not possible, as the species largely differed in their growth dynamics, especially between the coldest and the warmest chamber. We were thus unable to collect leaves of all the species in the same developmental stage from all the growth chambers simultaneously.

Leaf palatability was tested in multiple-choice feeding experiments, allowing us to compare palatability of leaves of different origins (temperature regime or species). Such experiments have been successfully used to compare palatability of plants of different origins in a range of previous studies (e.g., ^38–41^). As the herbivore, we used desert locust (*Schistocerca gregaria* Forskål; Orthoptera, Acrididae) individuals of 1–2 cm that were purchased from a commercial insect provider (www.sarancata.cz). The desert locust is a generalist herbivore occurring in many regions of Africa, the Middle East and Asia^42^. It is an extremely polyphagous leaf-chewing invertebrate, which makes it an excellent bioassay species for comparing leaf palatability across a wide range of plant species^43,44^. It does not occur in our study localities, and none of our model species thus has any history of co-evolution with this herbivore^45^.

The experiments were performed in circular arenas, 50 cm in diameter and 30 cm in height (in total, we used 80 arenas, as explained below). One-third of each arena was filled with common garden soil. Three or six tubes (see below) without lids, 1.5 ml each, were placed regularly into a circle inside the arena, with each tube approximately 10 cm from the edge. The tubes were inserted into the soil so that their top was approximately 1 mm above the soil surface and were filled with water. One randomly chosen fully expanded leaf from the upper part of the stem (for details see below) was placed into each tube with the petiole submerged in the water and the rest sticking out. We used leaves which were neither too young (i.e. fully developed) nor too old (i.e., without any signs of senescence). Five desert locust individuals were placed in the centre of each arena, and the whole arena was covered with a fine mesh to prevent the herbivores from escaping^21^. The experiments lasted 2–3 days, and the arenas were kept at room temperature (20–23 °C). The duration of each experiment was based on the actual leaf damage during each day of the experiment. The experiment was terminated when more than 80% of at least one leaf within the arena was consumed or when more than 2/3 of the leaves had visual damage^41^. Leaf palatability was recorded as the proportion of leaf area eaten by the herbivores separately for each leaf within each arena as described below. The desert locusts were used just once and were fed with leaves of *Taraxacum* sect. *Ruderalia* before the experiments, thus ensuring that none of the herbivores had prior exposure to *Impatiens* species^46^. Moreover, the herbivores did not receive any food 24 h prior to the experiments.

#### Experiment 1

In the first experiment, we compared the palatability of *Impatiens* leaves cultivated under three temperature regimes. In each arena, there were three leaves of the same *Impatiens* species, with each leaf originating from a different temperature regime (cold, warm and warm2050 regime in the growth chamber—see “Plant cultivation” section for details). Each plant species was studied in 10 arenas (10 replicates). There were 10 arenas per six *Impatiens* species, i.e., 60 arenas in total. The leaves were sampled when the plants of the particular species were already mature and flowering in all the growth chambers (June–July 2018).

#### Experiment 2

For the second experiment, we aimed to compare all the species within single experimental arenas. This allowed us more directly compare palatability among the six *Impatiens* species. For this experiment, we used leaves from plants cultivated in the cold growth chamber temperature regime used also in “Experiment 1” and from a common garden environment where the plants had more natural conditions (natural light and temperature conditions, more space). We selected these two environments because we were able to collect all the species in the same developmental stage at the same time in these two environments (plants were very desynchronized in the warm chambers). In the cold growth chamber temperature regime, leaves were collected and palatability tests were performed in July 2018. In the common garden, leaves were collected and palatability tests were performed in September 2018 when the plants were mature and flowering, i.e., the same phenological phase as in the growth chambers. In each arena, there were six leaves from different *Impatiens* species. We used 10 arenas for each environment, i.e., 20 replicates (arenas) in total.

### Leaf palatability and leaf trait measurements

Leaf palatability was recorded as the proportion of leaf area eaten by the herbivores. Fresh leaves were individually weighed and scanned both before and after herbivory (e.g., ^41^). Leaf area was estimated using ImageJ software (version 1.52a, Java 1.8.0_112, Wayen Rasband, U.S. National Institutes of Health, Bethesda, MD, USA; website: https://rsb.info.nih.gov/ij/download.html). After herbivory, the leaves were dried to a constant weight at 60 °C and weighed again. This information, together with information on leaf area, was used to calculate the specific leaf area (SLA; mm^2^ mg^−1^ dry mass) and leaf dry matter content (LDMC; mg dry mass g^−1^ fresh mass) for each leaf. The leaves were eaten evenly by the herbivores, and no leaf parts (such as leaf veins) were preferred or avoided. In two cases, the whole leaf was eaten. Then, we used the mean SLA and LDMC values of the other nine leaves from the respective temperature regime and species. We also analysed content of total carbon (C), nitrogen (N) and phosphorus (P) and calculated C:N, N:P and C:P ratios. Since it was not possible to analyse the leaf nutrient content in the leaves used directly for the feeding experiments due their small size, we used a mixed sample of ten randomly chosen leaves per species and growth chamber/common garden that were not exposed to the herbivores. The chemical analyses were performed in the Analytical laboratory of the Institute of Botany, Czech Academy of Sciences, Průhonice. The contents of nitrogen and carbon were analysed following Ehrenberger et Gorbach^47^. The content of phosphorus was analysed following Olsen et Dean^48^. These traits were chosen because they encompass a range of mechanical and chemical properties that are quantifiable within and between plant species and have been previously shown to be significantly related to leaf palatability^49^.

### Data analyses

Data from the two experiments were analysed separately using R software version 3.6.2^50^. Leaf palatability was square root-transformed, and SLA, LDMC and initial leaf area were log-transformed to meet test assumptions^51^.

#### Experiment 1

First, we tested the effects of temperature regime (growth chamber), species identity and their interaction on leaf palatability using a linear mixed effects model (LMM) with arena code as a random factor (random intercept) in R-package lmerTest^52^. Data on single leaves were used as a statistical unit. We repeated the test with initial leaf area as a covariate. However, the inclusion of the covariate did not affect the results in a significant way; thus, only the results without the covariate are presented (this is also true for Experiment 2). We also used the difference between the initial dry weight (recalculated using the initial and final leaf area and its final dry weight) and the final leaf dry weight instead of the proportion of eaten leaf area as the dependent variable in the tests. However, this approach did not bring any new insights. Moreover, leaf area and dry biomass were highly correlated (Pearson’s correlation coefficient r = 0.93, p < 0.001). These results are thus not presented.

Second, we tested how the leaf traits (SLA, LDMC, initial leaf area) were affected by temperature regime, species and their interaction using ANOVA. The differences in leaf nutrient content were not tested since these data were not replicated within the growth chambers and species due to a lack of available plant material for the analyses.

Further, we explored the effects of leaf traits and their interactions with temperature regime on leaf palatability after accounting for species identity and temperature regime using a LMM. For these tests, we used arena code and a code defining each species in each growth chamber as random factors. The latter was used to account for the fact that the traits measured within one species and temperature regime are not independent, and in the case of leaf nutrient content, they were only measured once. Since predictors of leaf palatability might be largely correlated, we selected a subset of uncorrelated factors based on a variance inflation factor (VIF) calculated with the ‘vifstep’ function in the R package usdm^53^. We considered variables with VIF values less than 3 as advised by Zuur et al.^54^ and this was done for morphological predictors (SLA, LDMC and leaf area) and predictors related to leaf nutrient content separately. This approach resulted in further use of all morphological predictors and the content of C, P and N:P ratio. First, we tested the effect of each predictor on leaf palatability separately comparing the models accounting for species identity and temperature regime with and without the tested predictor using AIC criteria. Predictors with significant effect on leaf palatability (ΔAIC > 1.5)^55^ were used for identification of optimal model using R package MuMIn^56^ with dredge function comparing all predictor combinations.

In all tests, the temperature regime was coded as a factor with three levels (cold, warm and warm2050 temperature regime), as the effects of temperature were not linear.

#### Experiment 2

The tests largely followed the logic described above for Experiment 1. First, we used a LMM with arena code as a random factor to test the effects of species identity, environment and their interaction on leaf palatability. Second, we tested how the leaf traits differed among the six *Impatiens* species and the two environments (growth chamber and common garden) using ANOVA. We also explored the effects of leaf traits and their interactions with the environment on leaf palatability after accounting for species identity and environment. The optimal model explaining leaf palatability was constructed in the same way as in Experiment 1.

## Results

### Effects of temperature and species identity on leaf palatability

**Figure 1:**
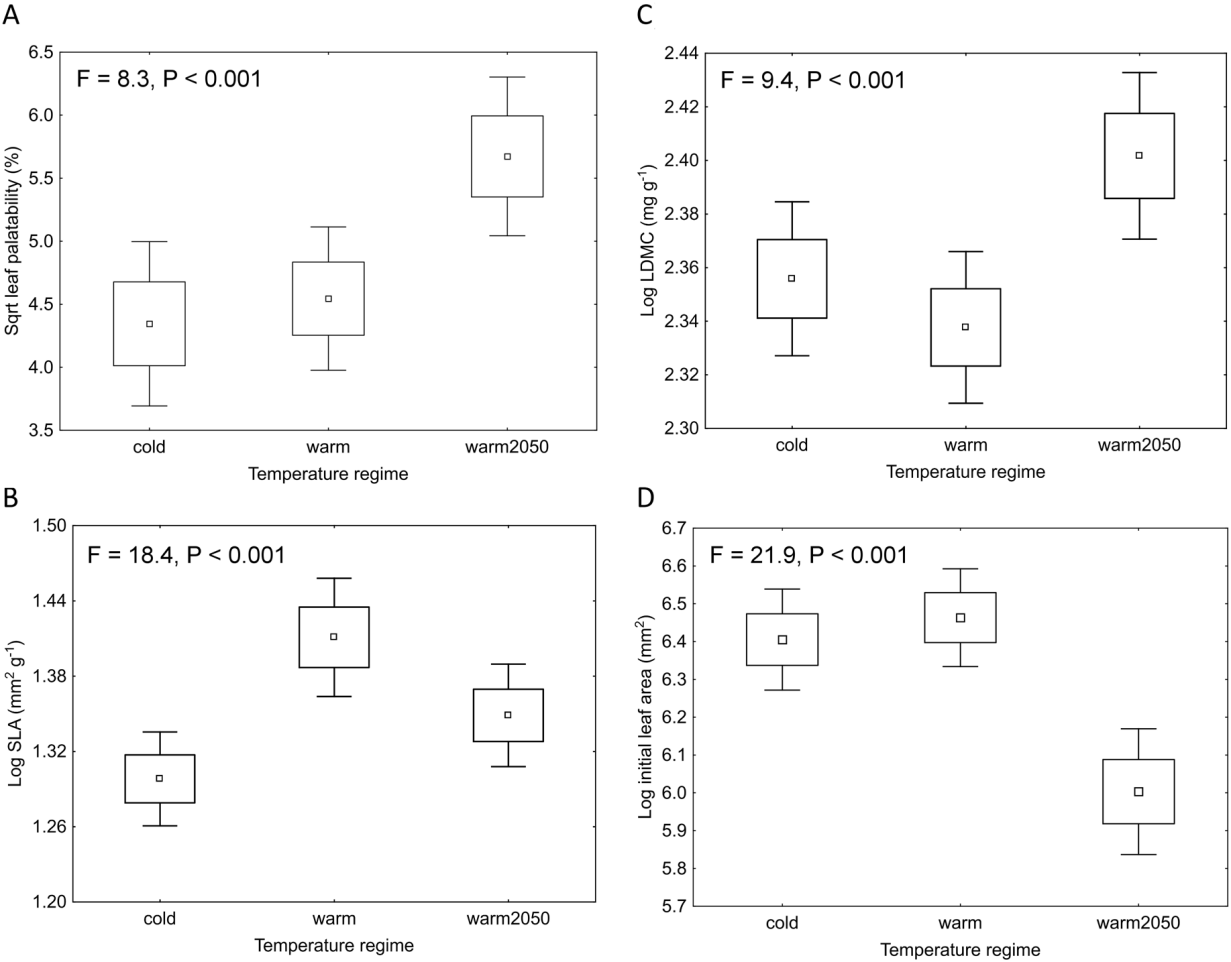
Effect of temperature regime on (**A**) leaf palatability, (**B**) SLA (specific leaf area), (**C**) LDMC (leaf dry matter content), and (**D**) initial leaf area in “Experiment 1” comparing leaf palatability and plant traits among the three temperature regimes in the growth chambers. P-values indicate significant differences among the three temperature regimes based on a linear mixed effects model with arena code as a random factor (**A**) and ANOVA (**B**–**D**). Only the differences among the temperature regimes are presented in this figure. Box plots show means, SE and 1.96*SE.

The results of Experiment 1 showed that leaf palatability differed among the plants cultivated in the three temperature regimes (LMM: F = 8.3, P < 0.001, Fig. 1A) and among the six *Impatiens* species (LMM: F = 6.5, P < 0.001, Supplementary Table S2). Moreover, there was a significant interaction between species and temperature regime (LMM: F = 2.6, P = 0.007, Supplementary Fig. S2A, for result details see Supplementary Table S3A). Leaves from the warm2050 regime were the most eaten (on average 38.0% of each leaf was eaten), and leaves from the cold regime were the least eaten in most of the studied species (25.1%). The only exception was *I. balsamina,* which had the highest herbivore damage of leaves from the cold regime (50% leaf area eaten in the cold regime compared to 34% and 43% in warm and warm2050 regime, respectively; Supplementary Fig. S2A).

**Figure 2:**
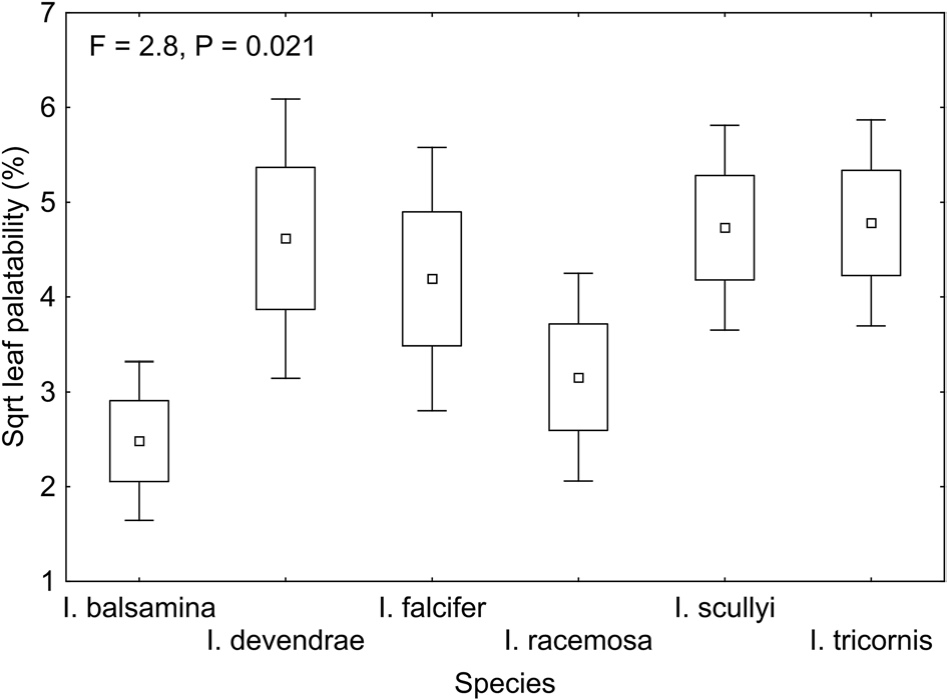
Differences in leaf palatability among six *Impatiens* species cultivated in the growth chamber and the common garden in “Experiment 2”. P-value indicates significant differences among the six species based on a linear mixed effects model with arena code as a random factor. Box plots show means, SE and 1.96*SE.

In Experiment 2, directly comparing the leaf palatability of the six different *Impatiens* species, leaf palatability also differed among the species (LMM: F = 2.8, P = 0.021) but was not affected by the environment (LMM: F = 1.8, P = 0.194) or the interaction between species and environment (LMM: F = 1.4, P = 0.236, for result details see Supplementary Table S3B). *I. balsamina* and *I. racemosa* tended to be the least damaged by the herbivores (9.6% and 15.9% leaf area eaten, respectively, compared to other species eaten on average 27.1–32.0%; Fig. 2, Supplementary Table S5, Supplementary Table S3A).

### Effects of temperature and species identity on leaf traits

In Experiment 1, all the tested leaf traits significantly differed among the temperature regimes and species (ANOVA: F > 9.4, P < 0.002 in all cases; for result details see Supplementary Table S4A, Supplementary Table S2). The highest values of SLA (27.9 mm^2^ g^−1^) were recorded in the warm temperature regime, and the highest LDMC values (262.0 mg g^−1^) were recorded in the warm2050 temperature regime (Fig. 1B,C). The initial leaf area was similar in the cold and warm regimes and was the lowest in the warm2050 regime (700, 733 and 513 mm^2^, respectively; Fig. 1D). However, a significant interaction between temperature regime and species identity in all the tested traits indicated different trends among the six species (Supplementary Table S2, Supplementary Fig. S2B–D). Leaf nutrient contents (C, P, N:P) did not show any consistent trend among temperature regimes across the studied species (Supplementary Table S2, Supplementary Table S5).

In Experiment 2, the six *Impatiens* species significantly differed in SLA, LDMC and initial leaf area (ANOVA: F > 3.7, P < 0.005 in all cases; for result details see Supplementary Table S3B), and there were also significant differences between the two environments, i.e., the growth chamber and the common garden (ANOVA: F > 96.9, P < 0.001 in all cases). SLA values were 1.45 times higher in the common garden compared to the growth chamber. In contrary, LDMC values were 1.37 times higher in the growth chamber. The strongest differences between the two environments were recorded in leaf area with on average 3 times larger leaves of plants cultivated in the common garden compared to leaves in the growth chambers (Supplementary Fig. S3B–D). As in the first experiment, the significant effects of the interactions between environment and species indicated different trends in initial leaf areas (ANOVA: F = 14.4, P < 0.001) and SLA (ANOVA: F = 2.3, P = 0.048) among *Impatiens* species from the common garden and the growth chamber. The interaction between species and environment was not significant for LDMC (ANOVA: F = 0.6, P = 0.66, Supplementary Fig. S3B–D).

### Importance of leaf traits for leaf palatability

**Figure 3:**
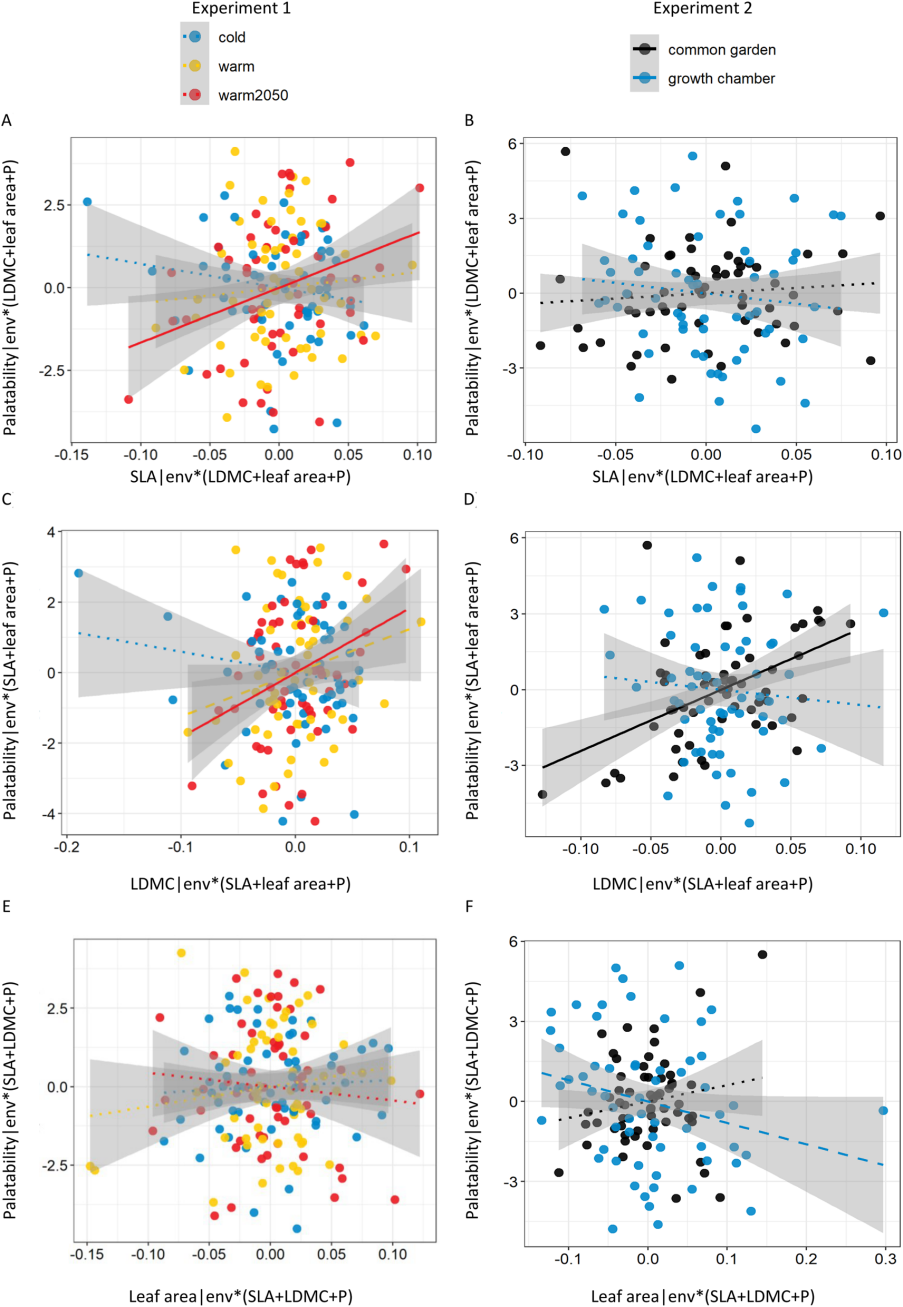
Relationship between leaf traits [SLA (specific leaf area), LDMC (leaf dry matter content) and initial leaf area] and leaf palatability using data from Experiment 1 (**A**,**B**,**C**) and Experiment 2 (**D**,**E**,**F**). Data on individual leaves are presented. Graph represents partial regression plot calculated from a linear mixed effects model where arena and a code defining each species in each environment (env) were used as random factors. The graph is showing the independent contribution of the variable on x axis (e.g., SLA in panel **A**) in explaining variation in palatability. The axes represent residuals of the models (e.g., *x*‐axis: SLA ~ env*(LDMC + leaf area + P);* y*‐axis: Palatability ~ env*(LDMC + leaf area + P) in panel **A**). Within each panel, we distinguished leaves from different growth chambers (cold, warm and warm2050 in Experiment 1; **A**,**B**,**C**) and different environments (common garden and cold growth chamber in Experiment 2; **D**,**E**,**F**). Lines were fitted for each growth chamber/environment to highlight the interaction between leaf traits and growth chamber/environment (see Table 1 for details). Lines indicate significance of the relationship (solid—P < 0.05, dashed—P < 0.1, dotted—non-significant).

In both Experiments 1 and 2, LDMC and initial leaf area significantly contributed to explaining leaf palatability. Herbivores generally preferred smaller leaves with higher LDMC values. Moreover, there were also significant pairwise interactions between temperature regime/environment and SLA and LDMC indicating that the relationships between leaf palatability and plant traits differed among the temperature regimes/environments (Table 1, Fig. 3, Supplementary Table S6, Supplementary Fig. S4). No variables related to leaf nutrient content significantly contributed to the explanation of variation in leaf palatability in any of the experiments (Table 1, Supplementary Table S6).

**Table 1:** Importance of leaf traits for explaining leaf palatability assessed by a linear mixed effects model.

	**Experiment 1**			**Experiment 2**		
	**NumDF**	**F**	**P**	**NumDF**	**F**	**P**
Species	5	1.42	0.329	5	0.47	0.783
Environment (Env)	**2**	**5.71**	0.013*	*1*	*6.23*	*0.053*
SLA	1	1.81	0.181	1	0.00	0.977
LDMC	**1**	**5.46**	0.021*	*1*	*3.47*	*0.065*
Leaf area	*1*	*3.49*	*0.065*	**1**	**6.55**	0.014*
Phosphorus (P)	1	1.95	0.220	1	0.65	0.576
Env:SLA	**2**	**5.78**	0.011*	*1*	*3.09*	*0.089*
Env:LDMC	**2**	**7.46**	0.002**	**1**	**10.72**	0.011*
Env:leaf area	2	2.06	0.134	1	0.00	0.990
Env:P	2	0.39	0.698	1	1.10	0.454

Arena and a combination of environment and species were used as random factors in all the tests. Leaf traits selected in the optimal model are presented. For details of leaf trait selection to the optimal model see Methods and Supplementary Table S6. Environment is represented by three different temperature regimes in the growth chambers in “Experiment 1” and by common garden vs. growth chamber in “Experiment 2”.

*SLA* specific leaf area, *LDMC* leaf dry matter content, *NumDF* degrees of freedom, *F* F value, *P* P-value.

P < 0.05 indicated by bold letters, P < 0.1 indicated by italics. **P < 0.01, *P < 0.05.

## Discussion

Global warming is predicted to significantly affect plant–herbivore interactions in higher altitudes and change the plant consumption rate, especially by ectotherm omnivores^6–8,10,57^. However, the indirect impacts of temperature (acting via changes in host plant quality) on herbivore performance have only rarely been studied so far (but see Descombes et al.^9^). We found a strong positive effect of temperature on leaf palatability that could be partly explained by changes in leaf morphology but not leaf chemistry. We also showed that the effects of elevated temperature on leaf palatability strongly differ among the *Impatiens* species. Conclusions about the effects of climate warming must thus be made specifically for each species.

### Effect of temperature on leaf palatability

The results of our study suggest that leaf palatability increases with temperature in most *Impatiens* species. Our results are in agreement with the general trend in nine most common woody host plant species of the generalist herbivore *Popillia japonica*^5^. Lemoine et al.^5^ suggests that temperature effects are species specific. In line with our results, Lemoine et al.^6^ also found that overall consumption rates increased with temperature in comparison of various 21 herbivore-plant pairs. However, there was substantial variation in thermal responses among individual herbivore-plant pairs depending not only on herbivore species but on host plant species as well. This conclusion was also confirmed in several other studies^9,10^. Lemoine et al.^6^ suggest this might be caused by differences in thermal optima of different herbivore species. Other studies on the effect of elevated temperature on plant palatability did not find any significant results in *Salix myrsinifolia*^58^ or *Quercus pubescens*^16^. However, these researchers noted that the reason for the lack of significance might be the very small temperature increase (only approximately 2 °C), which was not enough to affect the leaf traits which would then affect species palatability.

### Effect of temperature on leaf traits

We found that the temperature regime affected all the measured morphological leaf traits. First, we recorded unimodal relationship between temperature and SLA. This could be attributed to plants' ability to change leaf thickness or cell size depending on environmental conditions^59^. In our study, SLA increased when the temperature increased between the cold and warm growth chambers. In the growth chamber with elevated temperature based on the climate warming scenario (warm2050), SLA decreased again. Even though many studies have predicted a linear relationship between SLA and temperature (e.g., ^60–62^), studies looking at the response of individual species often show variable results. Loveys et al.^63^ reported nonlinear responses in SLA to temperature in several species, similar to the pattern detected in our study. This may be because the highest temperature is out of the species optimum, leading to changes in leaf structure^59^. LDMC showed a similar but inverse pattern to SLA, when LDMC positively responded to increased temperature in agreement with other studies^60,61,64^. While leaf area did not change between the cold and warm temperature regimes, it strongly decreased in the warmest (warm2050) temperature regime. This might be at least partly related to the nonlinear relationship between temperature and SLA or LDMC. Larger leaves are usually not advantageous at high temperatures because they increase transpiration^65^ (but see^66^). Surprisingly, we did not find any consistent effect of temperature on leaf nutrient content. This contrasts to Reich et Oleksyn^15^ and Zhang et al.^17^ demonstrating lower leaf nitrogen and phosphorus contents in plants growing in higher temperatures. *Impatiens* seems to respond to elevated temperatures by changing their leaf morphology while keeping the nutrient content in the leaves at a similar level. However, more data are needed to confirm this, as our leaf nutrient content data were not replicated and thus could not be formally tested. Note also that Münzbergová et al.^67^ demonstrated using the larger set of species and populations from the same model system that plants originating from different altitudes cultivated under standardized conditions showed that plants from higher altitude have higher nitrogen and phosphorus content than plants from lower altitudes. As in our study, Zvereva et Kozlov^57^ showed no effects of temperature increase on either nitrogen content or C/N ratios, and they suggested that not nutrients but defence chemicals are the main reason for the lower palatability of plants at elevated temperatures.

### Importance of leaf traits for leaf palatability

We demonstrated that herbivores prefer leaves with higher LDMC. This contrasts with the conclusions of several previous studies demonstrating that toughness is one of the most effective defences against herbivores^68,69^. However, our contrasting results are in concordance with other studies suggesting that the relationship between herbivory and LDMC might differ between different herbivore groups^9,70^. The capacity of an herbivore to ingest a plant species might be strongly limited and mediated by the mandibular force of the insect. Locusts (used in our study) have been shown to feed mostly on tough plants with high LDMC, while caterpillars mostly feed on plants with low LDMC^9,70^.

Herbivores in our study (locusts) also preferred smaller leaves. This is in contrast to many other studies that found that more vigorous plants suffered more leaf damage by range of insect herbivore groups (e.g., ^71,72^), as predicted by the Plant Vigour Hypothesis^73^. Similar to our study, Santos et al.^74^ found higher herbivory from gall-forming insects in smaller leaves. They suggested that smaller leaves should possess higher concentrations of resources that are essential for larval development. However, we found higher nitrogen and phosphorus contents (and less carbon) in the leaf biomass of larger leaves (Supplementary Fig. S5). The negative relationship between leaf area and the extent of herbivore damage might be due to an increase in the concentration of substances decreasing leaf palatability in larger leaves, as found by Albrectsen et al.^75^. They found that a decrease in palatability in willow seedlings with larger leaves was positively correlated with an increase in the condensed tannin concentration. Baskett et Schemske^76^ also suggested that young (i.e., small) leaves of *Phytolacca americana* may be more nutritious and less tough than the mature leaves, explaining their higher palatability.

Surprisingly, leaf nutrient contents did not contribute to explaining leaf palatability, as found by^9,20,77^. However, herbivore preferences are often driven not only by leaf nutrient contents but also by the production of specialized secondary metabolites^9,10,12^. Despite the review of Carmona et al.^78^, which tested the importance of plant traits for plant–herbivore interactions and concluded that morphological and physical traits are often more important for plant–herbivore interactions than chemical traits, the effect of chemical traits in our system cannot be excluded and requires further exploration.

### Different responses to elevated temperature among Impatiens species

Most *Impatiens* species in our study responded similarly to elevated temperature. The only exception was *I. balsamina*. In contrast to all the other species, *I. balsamina* increased SLA and decreased LDMC under the warmest (warm2050) temperature regime (Supplementary Fig. S2). Moreover, leaves of all other species were the most eaten when they were cultivated in the warmest temperature regime, while the leaves of *I. balsamina* were the most eaten when they were cultivated in the cold temperature regime. *I. balsamina* was also the least eaten species overall. However, this effect was only significant when the plants were cultivated in the common garden environment with higher temperatures, comparable to those in warmest regime in the growth chambers. When we compared the species cultivated in the cold temperature regime in the growth chamber, there were no differences in their palatability. The reason for the strong differences in the warmest regime might be that the original localities of all species included in our study are at approximately 2,500 m a. s. l., and the warm2050 regime represents the temperature under predicted climate change at 1,800 m a. s. l. (median of the lower altitudinal range of all *Impatiens* species in Nepal—see “Methods” for details). The only exception is *I. balsamina*, the seeds of which were collected at 1,330 m a. s. l., which is an altitude with temperatures simulated in the warm2050 regime in our study. *I. balsamina* thus seems to be well adapted to the temperatures of its home localities. Compared to other species, its leaves are tougher and might be better adapted to retaining water under elevated temperatures. Compared to those of other species, the leaves of *I. balsamina* are also less palatable to herbivores under elevated temperatures. Todd et al.^79^ recorded increased tannin concentrations in the leaves of *I. balsamina* following drought stress and suggested that tannins might play a role in its resistance to insect herbivores. While the other *Impatiens* species included in our study are expected to suffer from higher herbivory under elevated temperatures, the invasive potential of *I. balsamina* is higher under predicted global climate warming, as suggested by Tabak et von Wettberg^80^. The results thus indicate that increased palatability due to increased temperature may contribute to reduced performance in cold-adapted species under future climate warming, while at the same time, these changes in palatability may increase the success of warm-adapted species^81^. Invasion success of warm-adapted species might be further strengthened by strong effects on diversity of native communities as found for another *Impatiens* species^82,83^.

In the palatability comparison of *Impatiens* species (Experiment 2), all traits change in the same direction between the two tested environments (SLA and leaf area decreased and LDMC increased in the growth chamber compared to the common garden), but there is no consistent effect on leaf palatability. This suggests that other leaf traits not accounted for in this study are influencing leaf palatability. These may include defence chemicals, production of which requires further research as suggested by Zvereva et Kozlov^57^.

## Conclusion

The eastern Himalayas and Southeast Asia (e.g.,^84^), i.e., the regions of our study, are some of the biodiversity hotspots for the genus *Impatiens*. Its distribution covers wide altitudinal gradients as well as strong temperature gradients (differences in mean temperatures during growing season up to 25 °C). Most of the species, except warm adapted *I. balsamina*, became more palatable with increasing temperature, suggesting that cold adapted species will suffer higher herbivore pressure under warmer climates. These same species were also shown to suffer reduced germination under warmer conditions^34^, but increase their growth due to better function of photosynthetic apparatus^67^. Taken together, these results show that different aspects of plant life show different responses to climate, all these need to be considered to obtain an overall picture of species performance under warmer climate. At the same time, the results indicate that the effects of temperature are at least partly species-specific. Further studies on the drivers of among species differentiation in palatability and performance along climatic gradients are thus needed.

## Supplementary information

Supplementary file1 (DOCX 1514 kb)

## Data Availability

The data reported in this paper has been deposited at FigShare (https://doi.org/10.6084/m9.figshare.9438722.v1).
