# Supplementary material for: Plant palatability and trait responses to experimental warming
Source: Sci Rep. 2020 Jun 29;10:10526. doi: 10.1038/s41598-020-67437-0 (PMC7324391; doi:10.1038/s41598-020-67437-0)

*Journal:* Scientific Reports

**Plant palatability and trait responses to experimental warming**

Tomáš Dostálek^1,2,*^, Maan Bahadur Rokaya^2,3^, Zuzana Münzbergová^1,2^

^1^Institute of Botany, the Czech Academy of Sciences, Zámek 1, CZ-252 43 Průhonice, Czech Republic

^2^Department of Botany, Faculty of Science, Charles University, Benátská 2, CZ-128 01 Prague, Czech Republic

^3^Department of Biodiversity Research, Global Change Research Centre, the Czech Academy of Sciences, Bělidla 4a, CZ-603 00 Brno, Czech Republic

*Corresponding author's email: [tomas.dostalek@ibot.cas.cz](mailto:tomas.dostalek@ibot.cas.cz)

**Supplementary Table S1** Description of the six *Impatiens* species used in the study and site of their collection. Locality name, GPS coordinates (WGS 84) and altitude are shown for localities where the seeds were collected in autumn 2017. Mean premonsoon temperatures were obtained from the WorldClim database (Hijmans et al. 2005) as the mean temperatures from March to June for particular localities in 1960-90. The premonsoon period represents the time when most *Impatiens* species germinate and start to grow. Information on plant height, species altitudinal range and habitat types are species characteristics obtained from the literature (Akiyama et al. 1991; Grey-Wilson 1991; Pusalkar et Singh 2010; Akiyama et Ohba 2016). Temp regime indicates which temperature regimes used in the experiments in the growth chambers correspond to the natural species temperature niche.

| Species | Locality name | GPS coordinates |  | Altitude  (m a.s.l.) | Mean premonsoon temperature (°C) | Plant height (cm) | Species altitudinal range  (m a.s.l.) | Temp regime | Habitat type |
| --- | --- | --- | --- | --- | --- | --- | --- | --- | --- |
|  |  | N | E |  |  |  |  |  |  |
| *I. balsamina* L. | Kirtipur, Kathmandu | 27.68377 | 85.28356 | 1330 | 20.4 | 60 | 200-1800 | warm  warm2050 | Widely naturalized and cultivated in Himalayan foothills. |
| *I. devendrae* Pusalkar* | Darchula | 29.89019 | 80.92719 | 2728 | 10.9 | 30-80 | 2400-3200 | cold  (warm) | Shaded or partly shaded, moist places in Rhododendron forests and along forest edges. |
| *I. falcifer* Hook.f. | Phulchowki, Lalitpur | 27.57744 | 85.39950 | 2499 | 15.2 | 10-50 | 2300-3200 | cold  (warm) | Montane forests and stream beds. |
| *I. racemosa* DC. | Chandragiri | 27.66517 | 85.20458 | 2525 | 14.2 | 10-60 | 1300-3900 | cold  warm  warm2050 | Broad-leaved forest, rocky places, path sides, stream margins. |
| *I. scullyi* Hook.f. | Chame, Manang | 28.55227 | 84.24152 | 2688 | 12.8 | 30-60 | 2000-3600 | cold  warm | Forest understories, thickets along riverbanks, shaded moist places. |
| *I. tricornis* Lindl. ** | Chame, Manang | 28.55227 | 84.24152 | 2688 | 12.8 | 30-80 | 1000-3600 | cold  warm  warm2050 | Forest understories, thickets along riverbanks, shaded moist places. |

* Proper species determination is still in progress. Herbarium specimens were collected, and the species identity is currently being clarified using molecular data.

** Before revision by (Akiyama et Ohba 2016), usually called *I. scabrida.*

References:

Akiyama S & Ohba H (2016) Studies of Impatiens (Balsaminaceae) of Nepal 3. Impatiens scabrida and Allied Species. Bull Natl Mus Nat Sci Ser B Bot 42:121–130

Akiyama S, Ohba H & Wakabayashi M (1991) Taxonomic notes of the East Himalayan species of Impatiens. Studies of Himalayan Impatiens (Balsaminaceae). In: Ohba H, Malla SM (eds) The Himalayan Plants 2. University of Tokyo Press, Tokyo, pp 66–94

Grey-Wilson C (1991) Balsaminaceae. In: Grierson AJC, Long DG (eds) Flora of Bhutan. Royal Botanic Garden, Edinburgh, UK, pp 82–104

Hijmans RJ, Cameron SE, Parra JL, et al (2005) Very high resolution interpolated climate surfaces for global land areas. Int J Climatol 25:1965–1978. doi: 10.1002/joc.1276

Pusalkar PK & Singh DK (2010) Three New Species of Impatiens (Balsaminaceae) from Western Himalaya, India. 55:11

**Supplementary Table S2** Leaf palatability, leaf traits and leaf nutrient contents under the three temperature regimes in the six *Impatiens* species in Experiment 1. The same letters in the *Sig* columns indicate non-significant differences among temperature regimes within species (P > 0.05) after post‐hoc Tukey's test. Mean values for leaf palatability and leaf traits are presented (n=10). SLA = specific leaf area, LDMC = leaf dry matter content. Differences in leaf nutrient content were not tested since these data were not replicated within growth chambers and species variants due to the lack of available plant material for analyses.

| Species | Temp regime | Leaf palat.  (%) | Sig | Initial leaf area  (mm^2^) | Sig | SLA  mm^2^ mg^-1^ | Sig | LDMC  mg g^-1^ | Sig | N  (%) | C  (%) | P  (%) | C:N | C:P | N:P |
| --- | --- | --- | --- | --- | --- | --- | --- | --- | --- | --- | --- | --- | --- | --- | --- |
| *I. balsamina* | cold | 50.0 | a | 616.3 | b | 17.0 | ab | 192.2 | a | 1.77 | 37.18 | 0.18 | 21.05 | 201.81 | 9.59 |
|  | warm | 34.3 | a | 486.3 | b | 12.8 | a | 288.6 | b | 1.24 | 37.65 | 0.16 | 30.31 | 237.74 | 7.84 |
|  | warm2050 | 43.1 | a | 325.4 | a | 20.9 | b | 207.1 | a | 1.65 | 38.30 | 0.19 | 23.24 | 197.10 | 8.48 |
| *I. racemosa* | cold | 15.4 | a | 616.1 | a | 35.0 | a | 183.4 | a | 1.16 | 39.09 | 0.16 | 33.84 | 249.81 | 7.38 |
|  | warm | 28.9 | b | 639.2 | a | 40.4 | a | 182.3 | a | 1.03 | 36.00 | 0.15 | 34.95 | 239.18 | 6.84 |
|  | warm2050 | 33.8 | b | 505.0 | a | 36.3 | a | 210.9 | a | 1.75 | 41.08 | 0.12 | 23.42 | 339.87 | 14.51 |
| *I. scullyi* | cold | 9.0 | a | 1471.6 | a | 19.7 | ab | 290.4 | a | 1.16 | 36.96 | 0.19 | 31.91 | 199.05 | 6.24 |
|  | warm | 14.1 | a | 1470.9 | a | 23.6 | b | 290.1 | a | 1.65 | 36.67 | 0.19 | 22.16 | 193.13 | 8.71 |
|  | warm2050 | 42.2 | b | 1323.4 | a | 17.8 | a | 373.7 | b | 1.73 | 37.86 | 0.19 | 21.83 | 199.10 | 9.12 |
| *I. tricornis* | cold | 28.4 | a | 534.8 | ab | 18.0 | a | 231.4 | a | 1.32 | 36.63 | 0.20 | 27.84 | 186.89 | 6.71 |
|  | warm | 25.2 | a | 773.6 | b | 28.6 | c | 206.5 | a | 1.97 | 37.54 | 0.27 | 19.02 | 139.72 | 7.35 |
|  | warm2050 | 39.4 | a | 385.9 | a | 22.5 | b | 247.2 | a | 1.40 | 38.02 | 0.12 | 27.14 | 327.28 | 12.06 |
| *I. falcifer* | cold | 39.1 | ab | 453.3 | b | 18.2 | a | 248.3 | b | 1.51 | 37.38 | 0.20 | 24.72 | 185.10 | 7.49 |
|  | warm | 25.2 | a | 428.1 | b | 30.6 | b | 190.1 | a | 1.06 | 37.51 | 0.14 | 35.36 | 261.19 | 7.39 |
|  | warm2050 | 49.0 | b | 188.9 | a | 16.6 | a | 315.0 | c | 1.24 | 37.34 | 0.13 | 30.16 | 290.38 | 9.63 |
| *I. devendrae* | cold | 3.6 | a | 738.2 | ab | 17.9 | a | 275.0 | b | 0.79 | 37.85 | 0.11 | 48.13 | 345.03 | 7.17 |
|  | warm | 21.0 | b | 822.1 | b | 30.0 | b | 209.1 | a | 1.02 | 37.71 | 0.23 | 36.86 | 166.40 | 4.51 |
|  | warm2050 | 21.6 | b | 592.5 | a | 26.7 | b | 251.3 | b | 1.19 | 38.71 | 0.17 | 32.48 | 224.06 | 6.90 |

**Supplementary Table S3** Details of the results from the tests of species, environment and their interaction on leaf palatability using a linear mixed effects model. Environment is represented A) by three different temperature regimes in the growth chambers in Experiment 1 and B) by common garden vs. growth chamber in Experiment 2. Arena code was used as random factors in the tests. *** P < 0.001, ** P < 0.01, * P < 0.05

| A) Experiment 1 |  |  |  |  |  |  |  |
| --- | --- | --- | --- | --- | --- | --- | --- |
|  | Sum Sq | Mean Sq | NumDF | DenDF | F value | P |  |
| Species | 132.9 | 26.6 | 5 | 51 | 6.53 | 0.000 | *** |
| Environment | 67.6 | 33.8 | 2 | 102 | 8.31 | 0.000 | *** |
| Species:environment | 107.4 | 10.7 | 10 | 102 | 2.64 | 0.007 | ** |
|  |  |  |  |  |  |  |  |
| B) Experiment 2 |  |  |  |  |  |  |  |
|  | Sum Sq | Mean Sq | NumDF | DenDF | F value | P |  |
| Species | 91.6 | 18.3 | 5 | 90 | 2.82 | 0.021 | * |
| Environment | 11.8 | 11.8 | 1 | 18 | 1.82 | 0.194 |  |
| Species:environment | 45.0 | 9.0 | 5 | 90 | 1.39 | 0.236 |  |

**Supplementary Table S4** Details of the results from the tests of species, environment and their interaction on leaf traits (SLA, LDMC and initial leaf area) using ANOVA. Environment is represented A) by three different temperature regimes in the growth chambers in Experiment 1 and B) by common garden vs. growth chamber in Experiment 2. SLA = specific leaf area, LDMC = leaf dry matter content. *** P < 0.001, ** P < 0.01, * P < 0.05, . P < 0.1

| A) Experiment 1 |  |  |  |  |  |  |  | B) Experiment 2 | | |  |  | |  |
| --- | --- | --- | --- | --- | --- | --- | --- | --- | --- | --- | --- | --- | --- | --- |
|  |  |  |  |  |  |  |  |  |  |  |  |  |  | |
| Response: SLA |  |  |  |  |  |  |  |  |  |  |  |  |  | |
|  | Df | Sum Sq | Mean Sq | F value | P |  |  | Df | Sum Sq | Mean Sq | F value | P |  | |
| Species | 5 | 2.13 | 0.43 | 43.2 | 0.000 | *** |  | 5 | 0.83 | 0.17 | 3.7 | 0.004 | ** | |
| Environment | 2 | 0.36 | 0.18 | 18.4 | 0.000 | *** |  | 1 | 4.30 | 4.30 | 96.9 | 0.000 | *** | |
| Species:Environment | 10 | 0.75 | 0.08 | 7.6 | 0.000 | *** |  | 5 | 0.52 | 0.10 | 2.3 | 0.048 | * | |
| Residuals | 153 | 1.51 | 0.01 |  |  |  |  | 108 | 4.79 | 0.04 |  |  |  | |
|  |  |  |  |  |  |  |  |  |  |  |  |  |  | |
| Response: LDMC |  |  |  |  |  |  |  |  |  |  |  |  |  | |
|  | Df | Sum Sq | Mean Sq | F value | P |  |  | Df | Sum Sq | Mean Sq | F value | P |  | |
| Species | 5 | 0.63 | 0.13 | 19.1 | 0.000 | *** |  | 5 | 5.35 | 1.07 | 33.7 | 0.000 | *** | |
| Environment | 2 | 0.12 | 0.06 | 9.4 | 0.000 | *** |  | 1 | 3.33 | 3.33 | 105.0 | 0.000 | *** | |
| Species:Environment | 10 | 0.52 | 0.05 | 7.9 | 0.000 | *** |  | 5 | 0.10 | 0.02 | 0.6 | 0.663 |  | |
| Residuals | 153 | 1.01 | 0.01 |  |  |  |  | 108 | 3.42 | 0.03 |  |  |  | |
|  |  |  |  |  |  |  |  |  |  |  |  |  |  | |
| Response: initial leaf area | |  |  |  |  |  |  |  |  |  |  |  |  | |
|  | Df | Sum Sq | Mean Sq | F value | P |  |  | Df | Sum Sq | Mean Sq | F value | P |  | |
| Species | 5 | 4.50 | 0.90 | 29.2 | 0.000 | *** |  | 5 | 34.20 | 6.84 | 63.4 | 0.000 | *** | |
| Environment | 2 | 1.35 | 0.67 | 21.9 | 0.000 | *** |  | 1 | 33.11 | 33.11 | 307.1 | 0.000 | *** | |
| Species:Environment | 10 | 0.54 | 0.05 | 1.8 | 0.073 | . |  | 5 | 7.76 | 1.55 | 14.4 | 0.000 | *** | |
| Residuals | 153 | 4.71 | 0.03 |  |  |  |  | 108 | 11.65 | 0.11 |  |  |  | |

**Supplementary Table S5** Leaf palatability, leaf traits and leaf nutrient contents in the six *Impatiens* species in a common garden and a growth chamber environment in Experiment 2. The same letters indicate non-significant differences among the species within each environment (P > 0.05) after post‐hoc Tukey's test. Mean values for leaf palatability and leaf traits are presented (n=10). SLA = specific leaf area, LDMC = leaf dry matter content. Differences in leaf nutrient content were not tested since these data were not replicated within growth chambers and species variants due to the lack of available plant material for analyses.

| Species | Temp regime | Leaf palat.  (%) | Sig | Initial leaf area  (mm^2^) | Sig | SLA  mm^2^ mg^-1^ | Sig | LDMC  mg g^-1^ | Sig | N  (%) | C  (%) | P  (%) | C:N | C:P | N:P |
| --- | --- | --- | --- | --- | --- | --- | --- | --- | --- | --- | --- | --- | --- | --- | --- |
| *I. balsamina* | Garden | 3.6 | a | 1944.2 | d | 29.3 | a | 121.0 | a | 2.47 | 33.81 | 0.30 | 13.69 | 114.25 | 8.34 |
| *I. racemosa* |  | 17.7 | ab | 700.2 | b | 28.8 | a | 168.2 | b | 2.41 | 41.18 | 0.26 | 17.09 | 155.48 | 9.10 |
| *I. scullyi* |  | 15.4 | ab | 3282.6 | e | 29.8 | a | 240.3 | c | 2.39 | 36.72 | 0.25 | 15.39 | 146.95 | 9.55 |
| *I. tricornis* |  | 24.3 | ab | 2296.5 | de | 27.3 | a | 194.7 | b | 2.66 | 33.94 | 0.30 | 12.77 | 112.19 | 8.79 |
| *I. falcifer* |  | 22.5 | ab | 455.1 | a | 32.2 | a | 148.1 | b | 2.99 | 34.67 | 0.27 | 11.59 | 127.59 | 11.01 |
| *I. devendrae* |  | 36.4 | b | 1345.5 | c | 27.0 | a | 216.4 | c | 2.32 | 34.84 | 0.33 | 14.99 | 106.06 | 7.08 |
| *I. balsamina* | Growth chamber | 15.7 | a | 825.9 | d | 15.9 | a | 170.0 | a | 1.77 | 37.18 | 0.18 | 21.05 | 201.81 | 9.59 |
| *I. racemosa* |  | 14.0 | a | 518.8 | bc | 21.4 | bc | 245.0 | bc | 1.16 | 39.09 | 0.16 | 33.84 | 249.81 | 7.38 |
| *I. scullyi* |  | 41.0 | a | 847.6 | cd | 19.1 | c | 315.5 | d | 1.16 | 36.96 | 0.19 | 31.91 | 199.05 | 6.24 |
| *I. tricornis* |  | 33.1 | a | 341.9 | b | 23.3 | c | 243.7 | bd | 1.32 | 36.63 | 0.20 | 27.84 | 186.89 | 6.71 |
| *I. falcifer* |  | 31.7 | a | 191.0 | a | 22.4 | c | 221.6 | ab | 1.51 | 37.38 | 0.20 | 24.72 | 185.10 | 7.49 |
| *I. devendrae* |  | 27.7 | a | 568.4 | bd | 17.3 | b | 303.7 | cd | 0.79 | 37.85 | 0.11 | 48.13 | 345.03 | 7.17 |

**Supplementary Table S6** Leaf traits explaining leaf palatability using a linear mixed effects model after accounting for the effects of environment and species in the null model. Environment is represented A) by three different temperature regimes in the growth chambers in Experiment 1 and B) by common garden vs. growth chamber in Experiment 2. The values shown are values of ΔAIC comparing the model with the given trait to the null model. Numbers in the brackets show AIC values of the null model. Leaf traits significantly improving the model (decreasing model ΔAIC>1.5) are in bold. Arena and a code defining each species in each environment were used as random factors in all the tests. Asterisks indicate leaf traits included in the optimal model explaining leaf palatability. SLA = specific leaf area, LDMC = leaf dry matter content.

|  | A) Experiment 1 | B) Experiment 2 |
| --- | --- | --- |
|  | ΔAIC | ΔAIC |
| Null model (AIC) | 0 (761.9) | 0 (582.7) |
| C | 1.5 | 2.3 |
| N:P | 3.5 | 2.3 |
| **P** | **-5.0*** | **-7.4*** |
| **SLA** | **-1.8*** | **-6.8*** |
| **LDMC** | **-4.0*** | **-10.3*** |
| **Initial leaf area** | **-2.5*** | **-4.6*** |
| Environment × C | -0.4 | -0.4 |
| Environment × NP | -0.9 | 1.8 |
| **Environment × P** | **-18.9*** | **-15.1*** |
| **Environment × SLA** | **-7.3*** | **-11.1*** |
| **Environment × LDMC** | **-13.0*** | **-20.1*** |
| **Environment × initial leaf area** | **-7.4*** | **-5.9*** |
| Optimal model | **-61.3** | **-40.9** |

**Supplementary Figure S1** The courses of the temperatures during the days in the growth chambers. The same day length and radiation were used, i.e., 12 h of light (06.00–18.00 h; 250 μmol m^-2^ s^-1^) and 10 h of full dark with a gradual change in light availability in the transition between the light and dark period over 1 h.

**Supplementary Figure S2** Effect of temperature regime on A) leaf palatability, B) SLA (specific leaf area), C) LDMC (leaf dry matter content), and D) initial leaf area in multichoice feeding experiment categorized by the six *Impatiens* species in Experiment 1. Means and their standard errors are shown. *** P < 0.001, ** P < 0.01, * P < 0.05, . P < 0.1. P-values are based on the results of linear mixed effects model for leaf palatability (A) and on ANOVA for plant traits (B, C, D).

A)


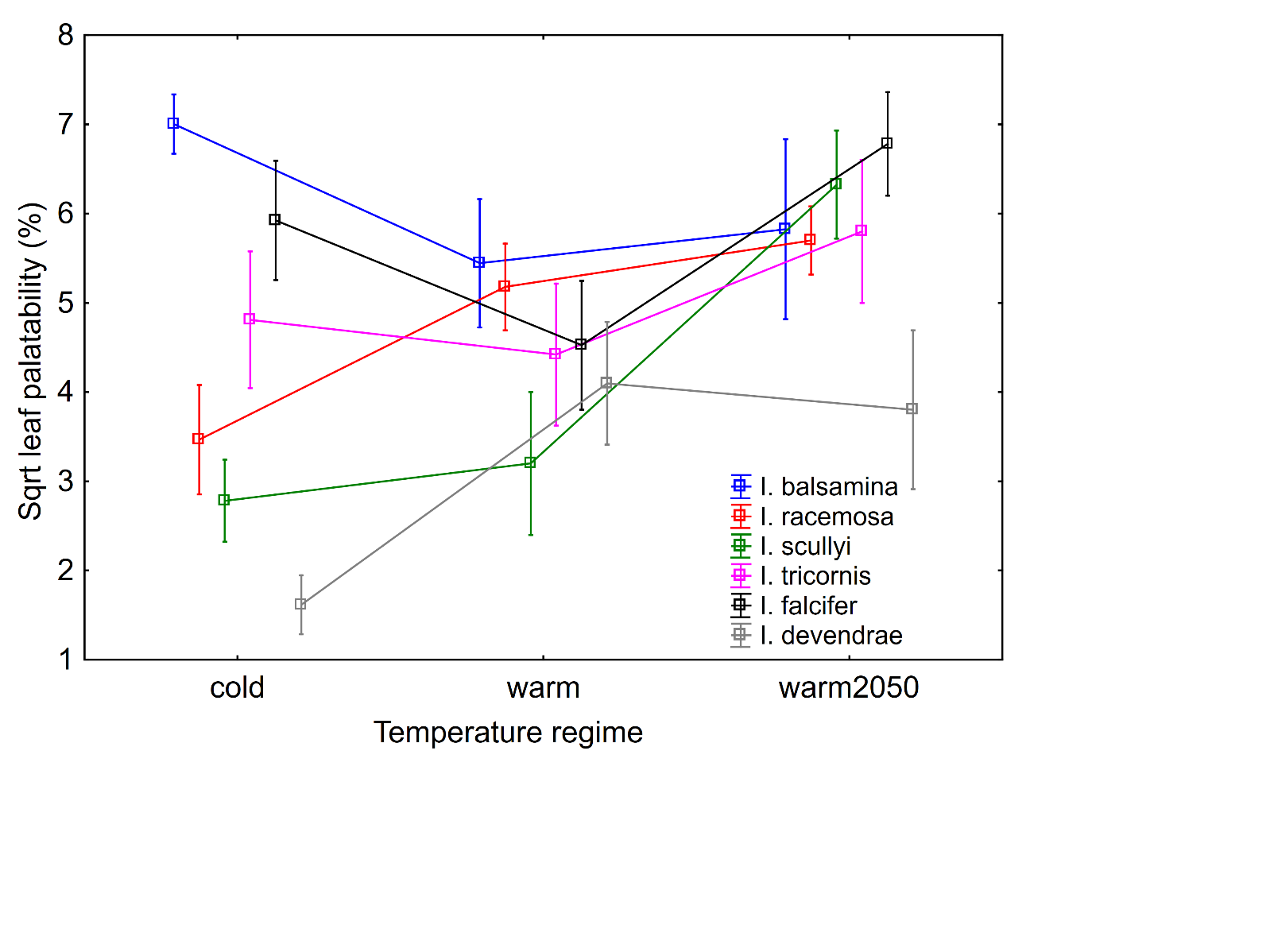


B)


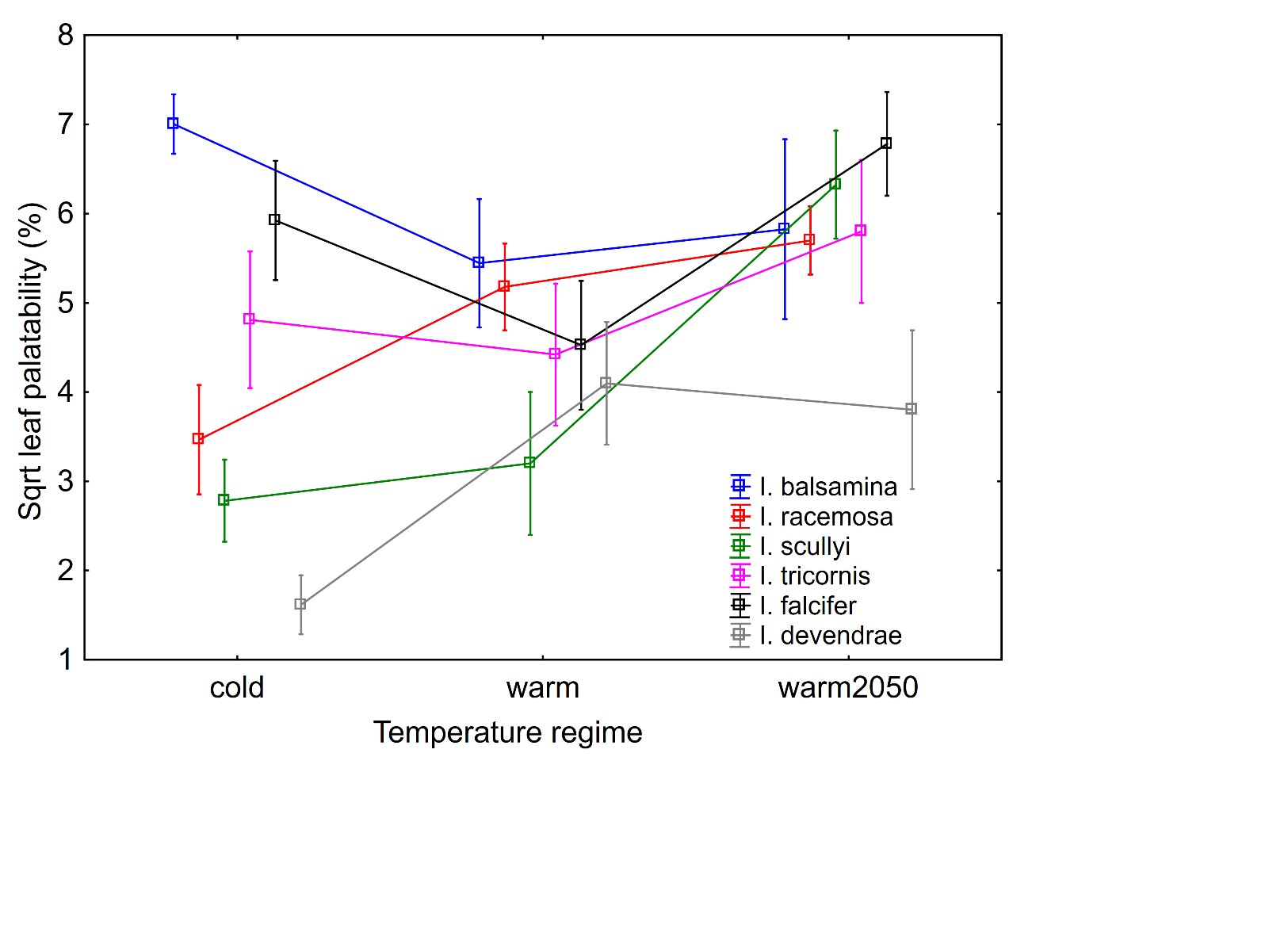

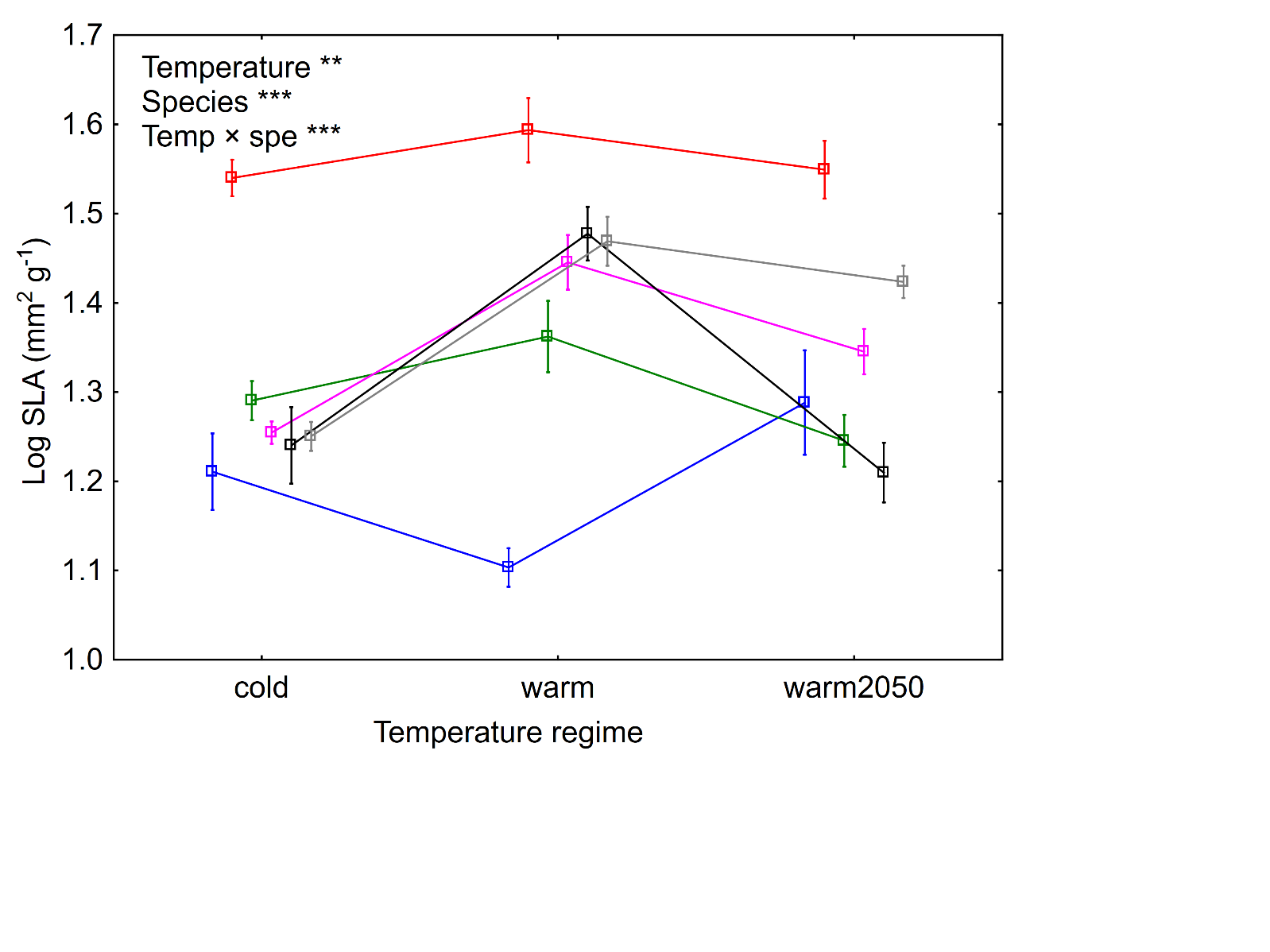


C)


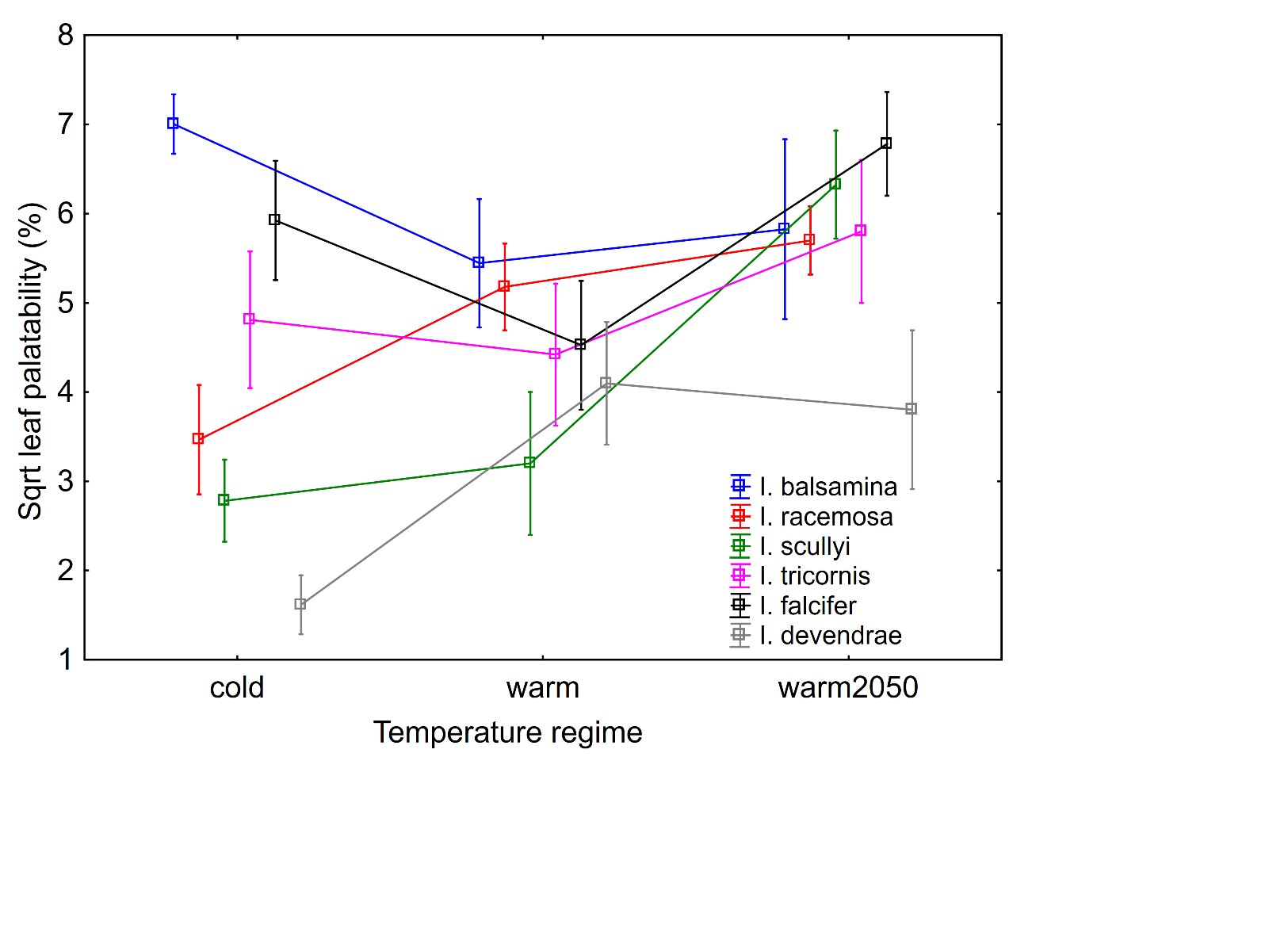

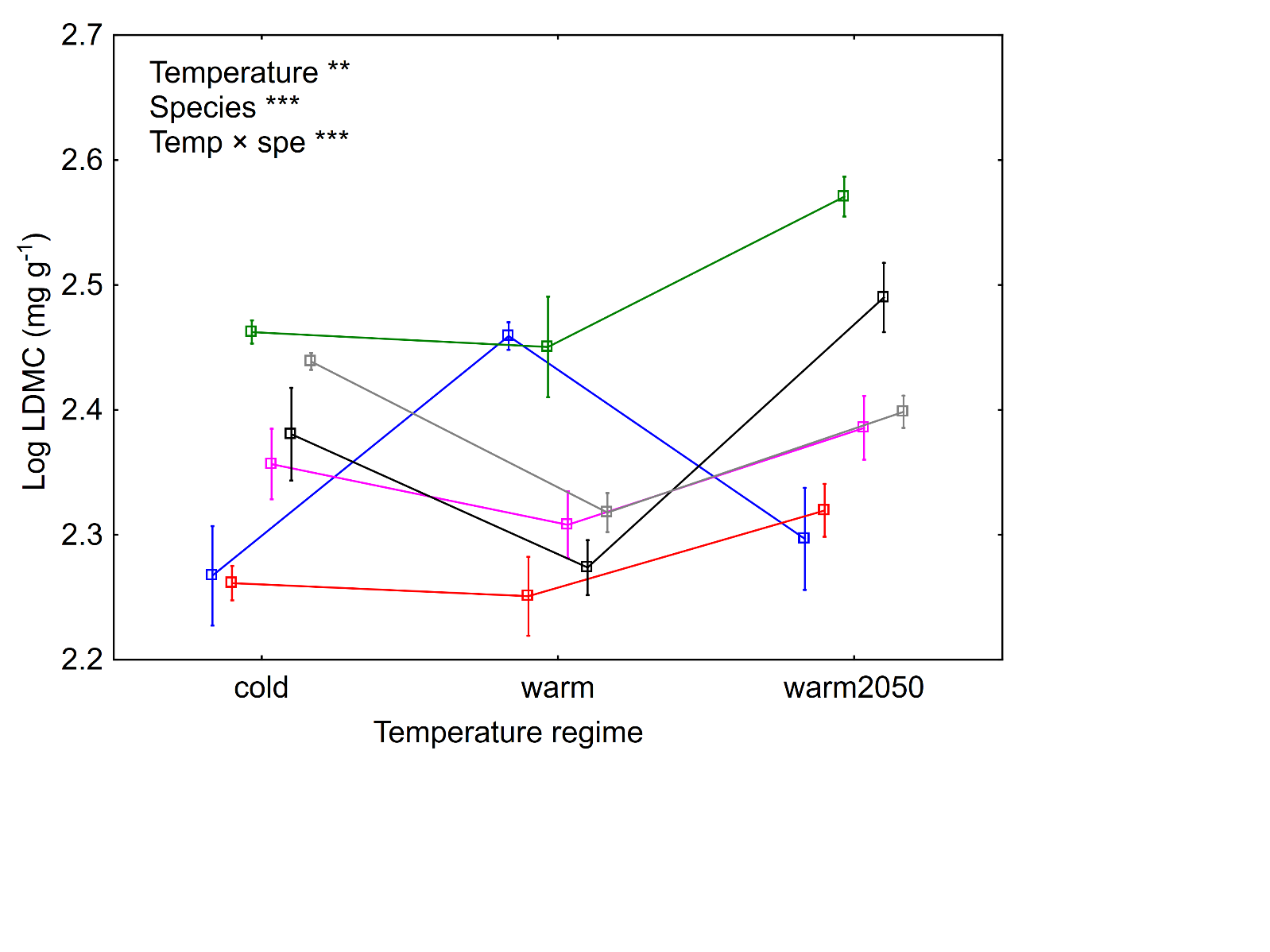


D)


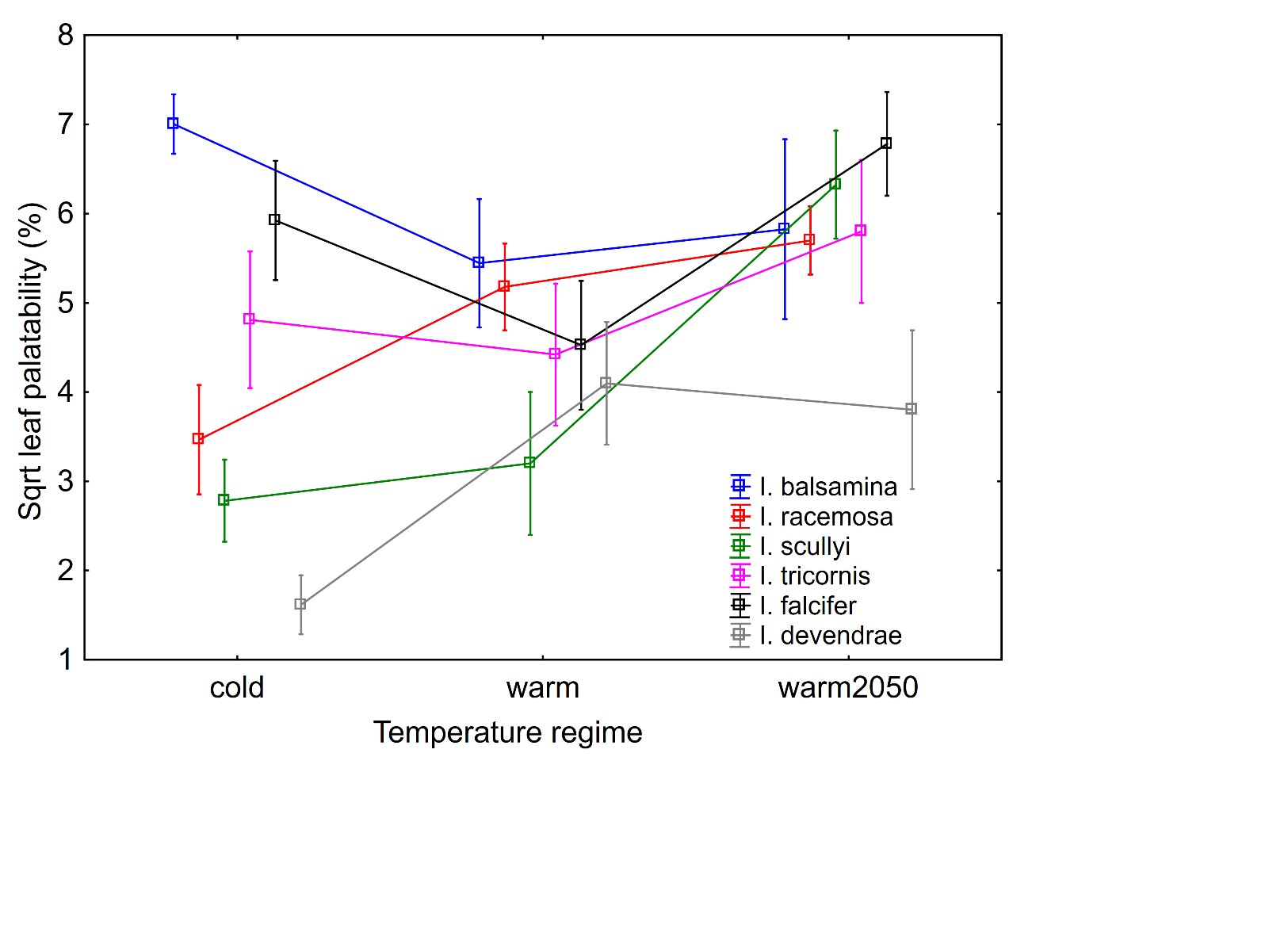

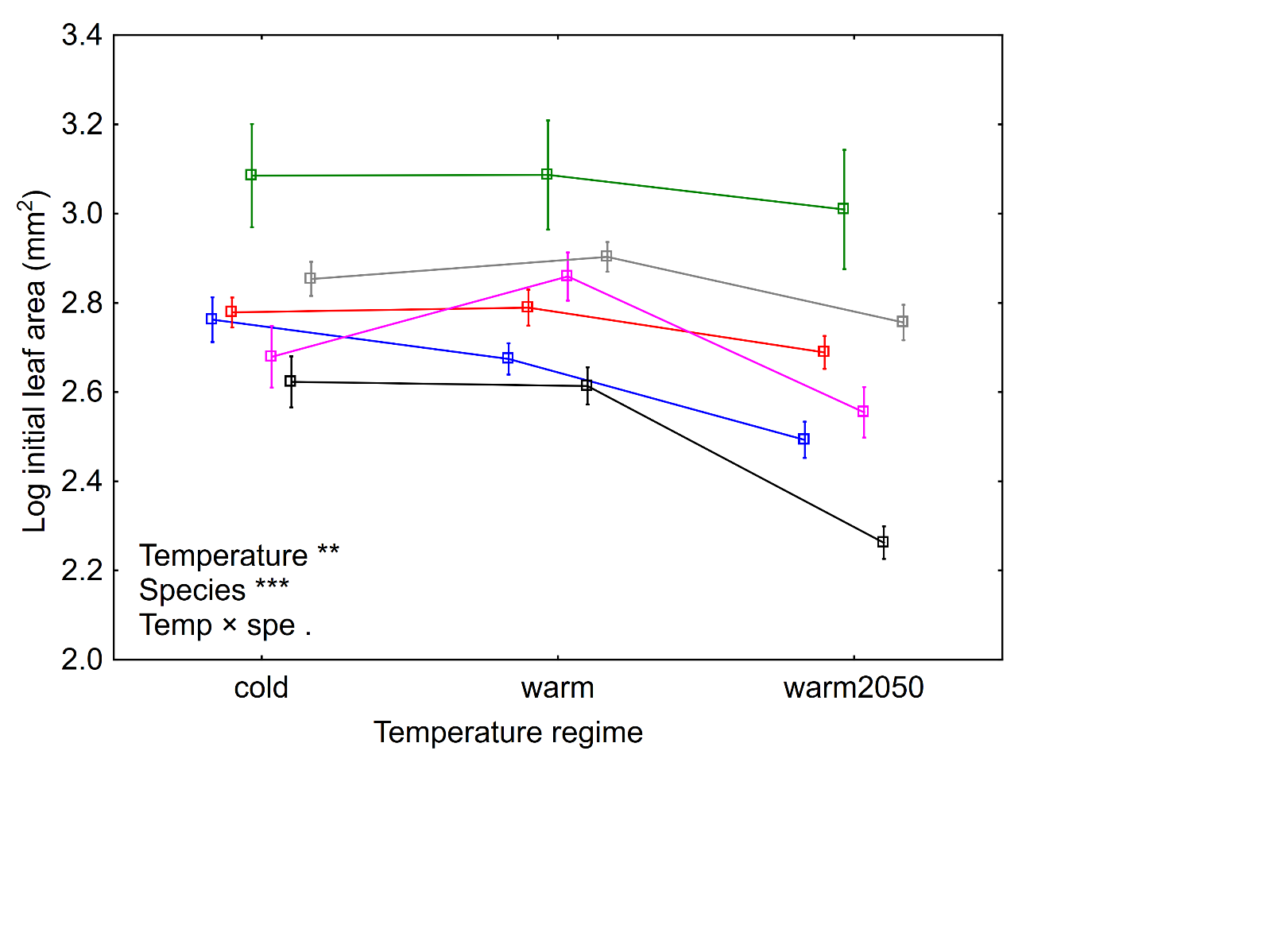


**Supplementary Figure S3** Differences in A) SLA (specific leaf area), B) LDMC (leaf dry matter content), and C) initial leaf area among six *Impatiens* species between the two environments (common garden vs. growth chamber) in Experiment 2. Means and their standard errors are shown. *** P < 0.001, ** P < 0.01, * P < 0.05, . P < 0.1, n.s. non-significant. P-values are based on the results of ANOVA test.

A)


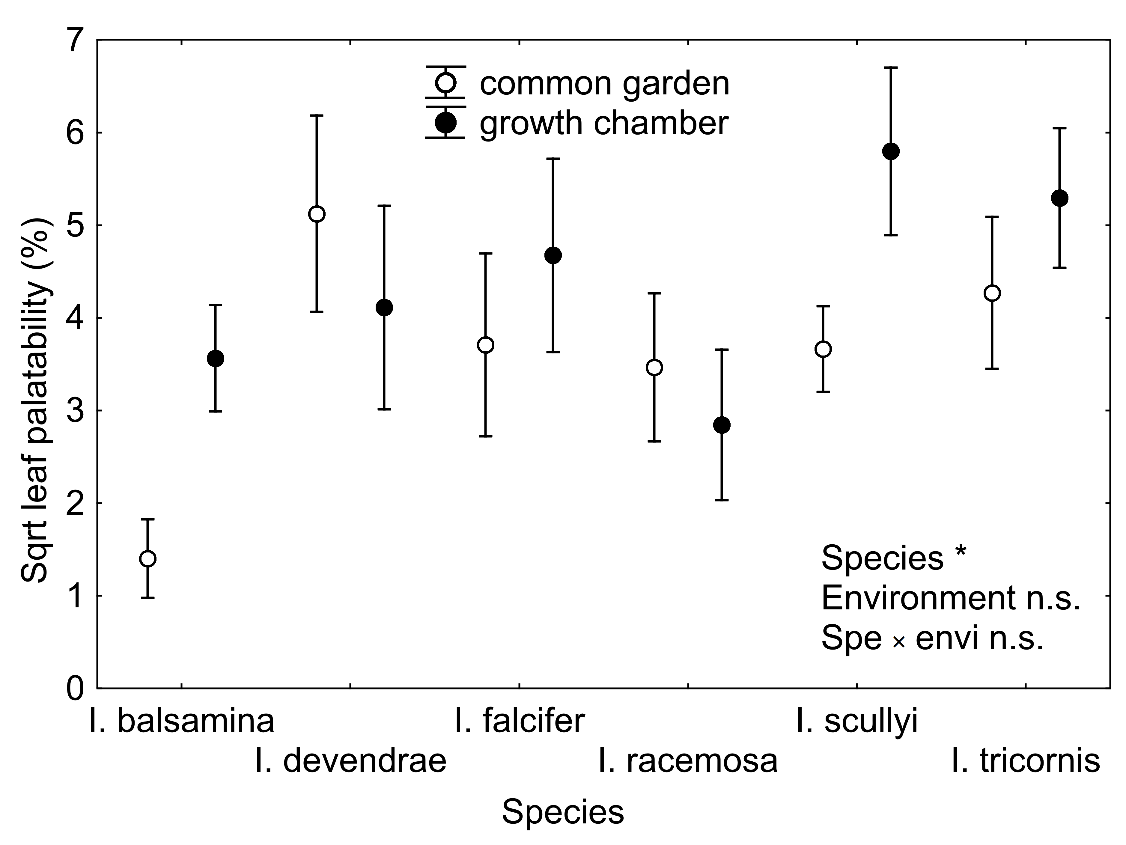


B)


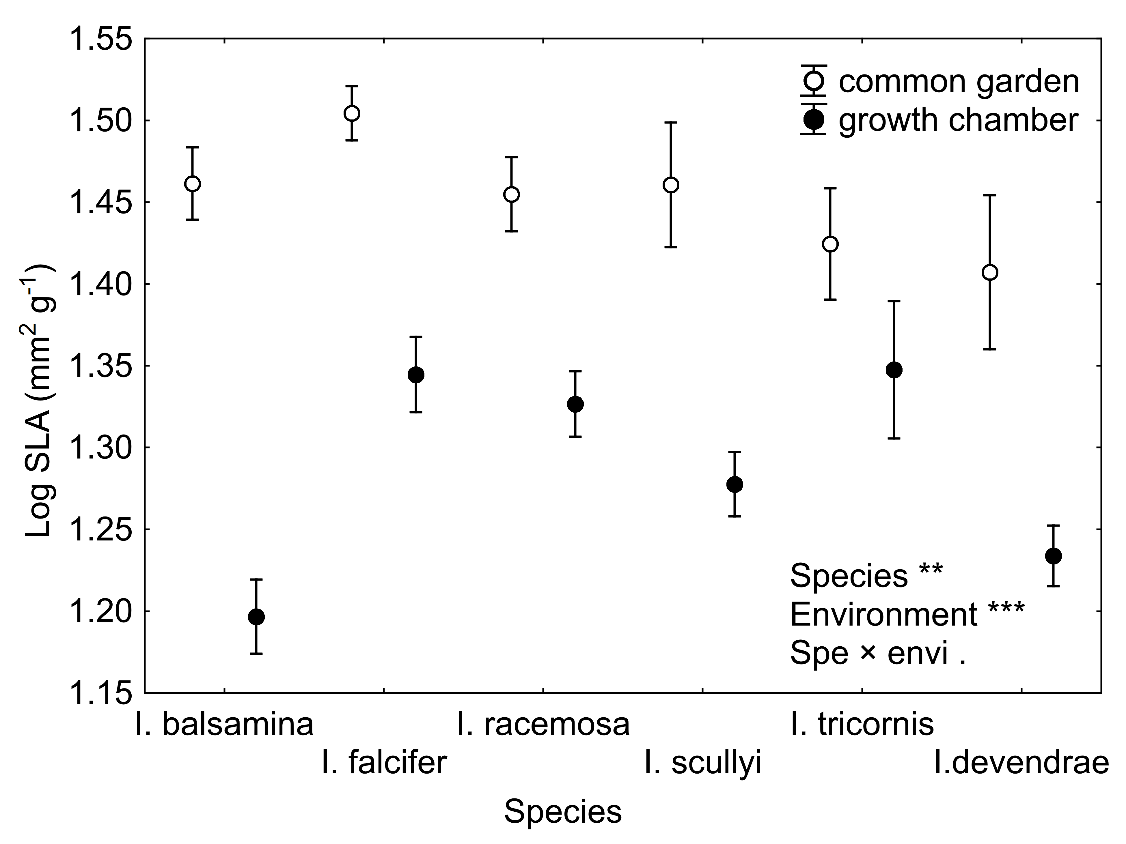


C)


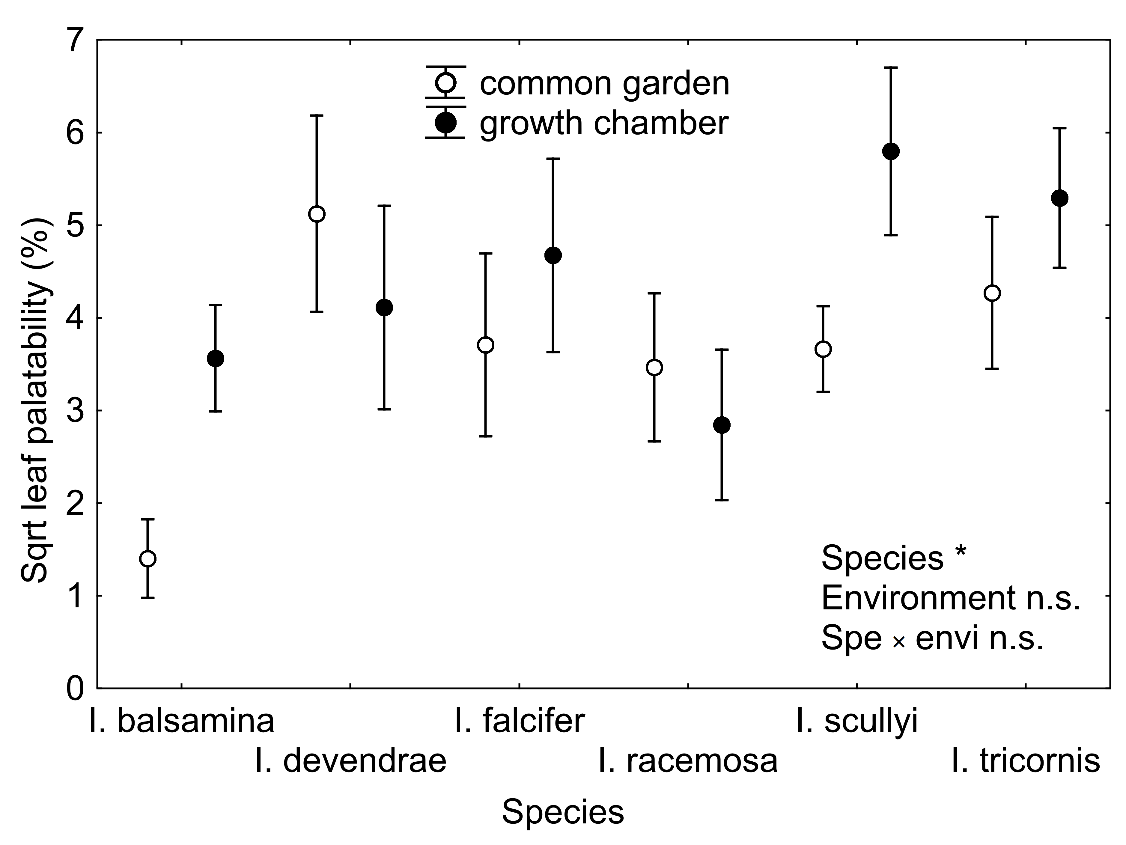

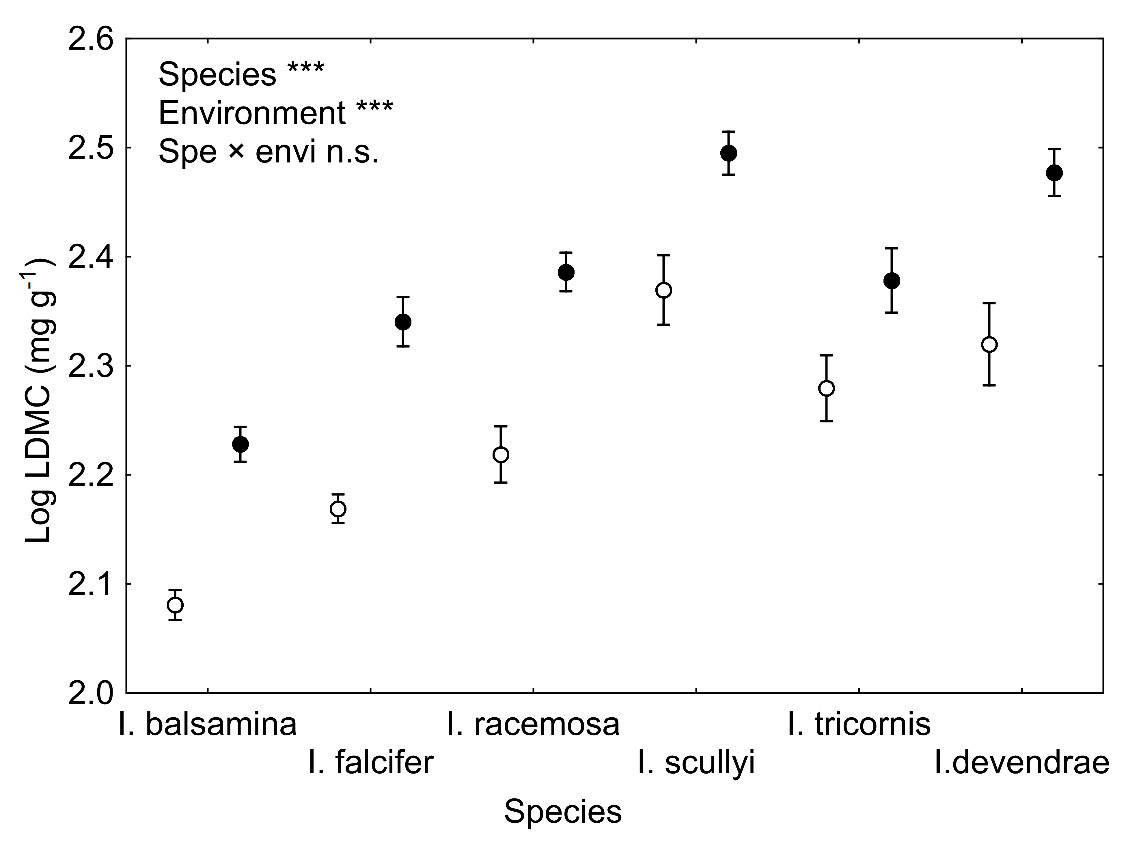


D)


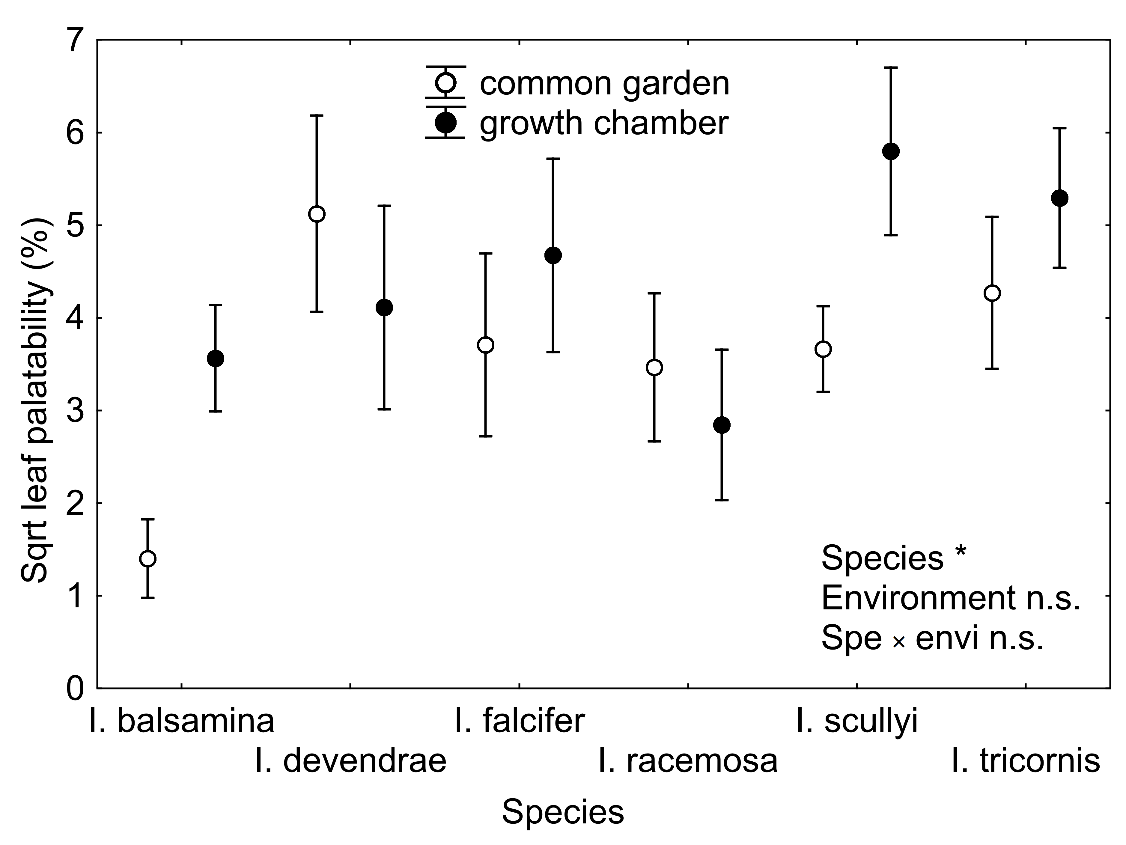

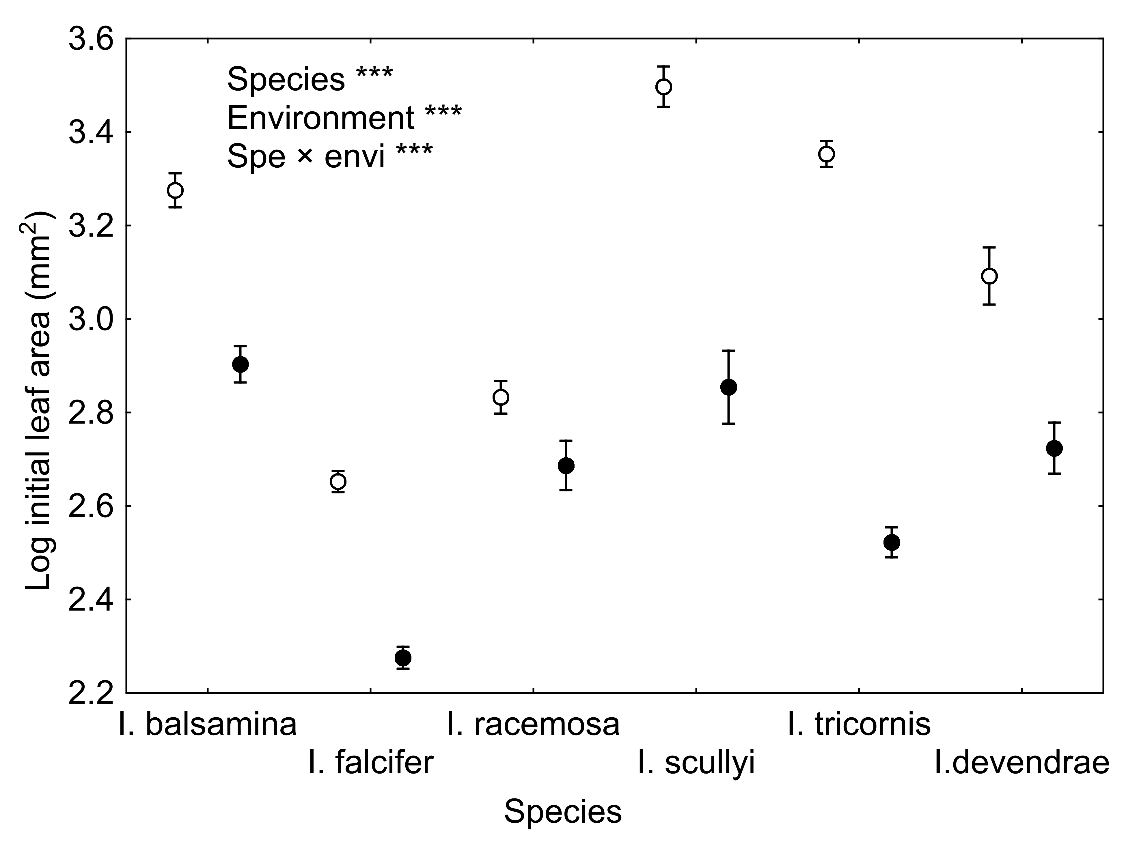


**Supplementary Figure S4** Relationship between leaf traits (SLA, LDMC and initial leaf area) and leaf palatability using data from Experiment 1 (A, B, C) and Experiment 2 (D, E, F). Data on individual leaves are presented. Within each panel, we distinguished leaves from different growth chambers (cold, warm and warm2050 in Experiment 1; A, B, C) and different environments (common garden and cold growth chamber in Experiment 2; D, E, F). Lines were fitted for each growth chamber/environment to highlight the interaction between leaf traits and leaf palatability (see Table 1 for details). Lines indicate the significance of the relationship (solid - P < 0.05, dashed - P<0.1, dotted - non-significant). SLA = specific leaf area, LDMC = leaf dry matter content.

**
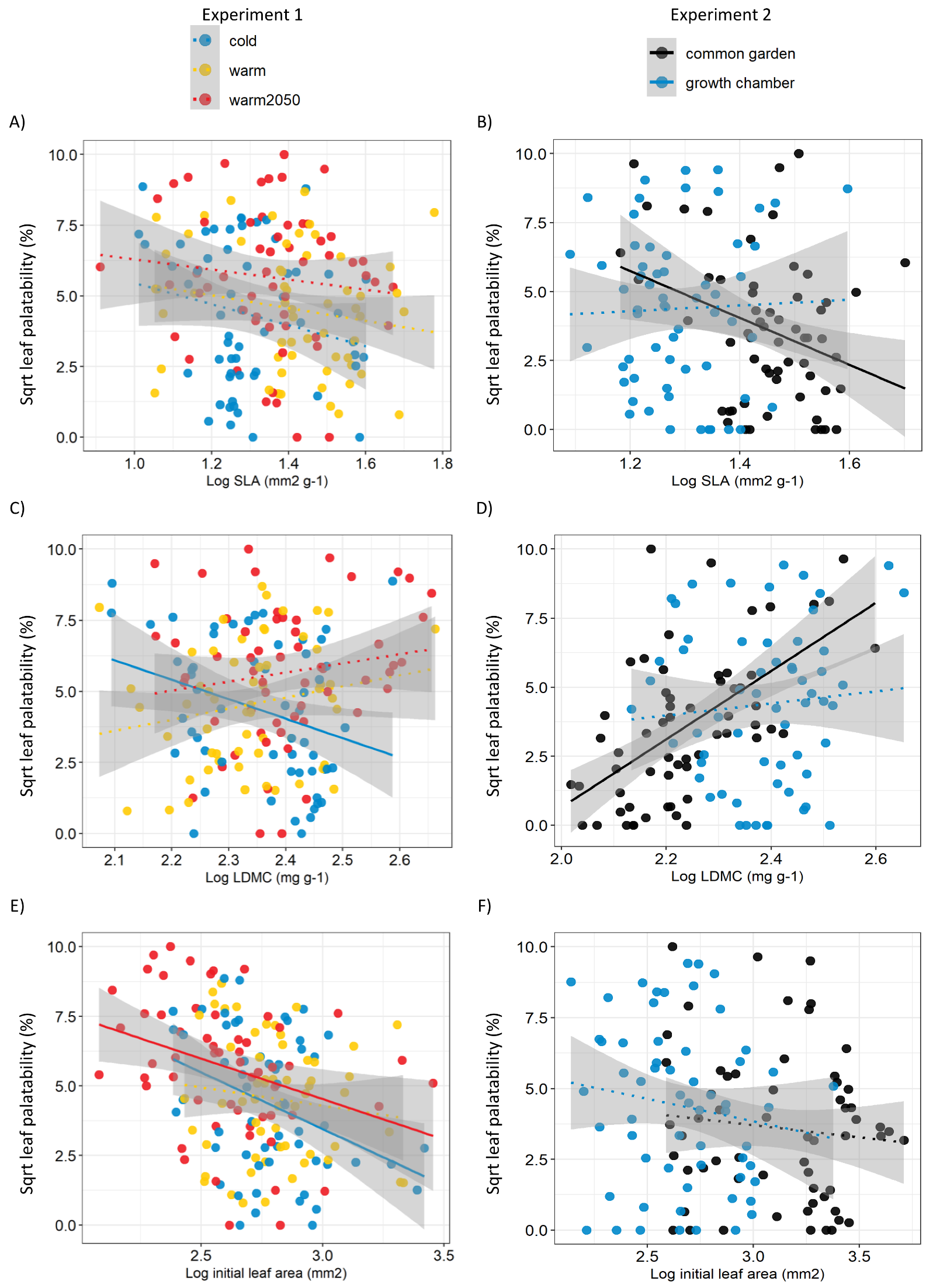
**

**Supplementary Figure S5** Relationship between leaf size and its nutrient content recorded at leaves both in Experiment 1 and 2 tested by linear regression.


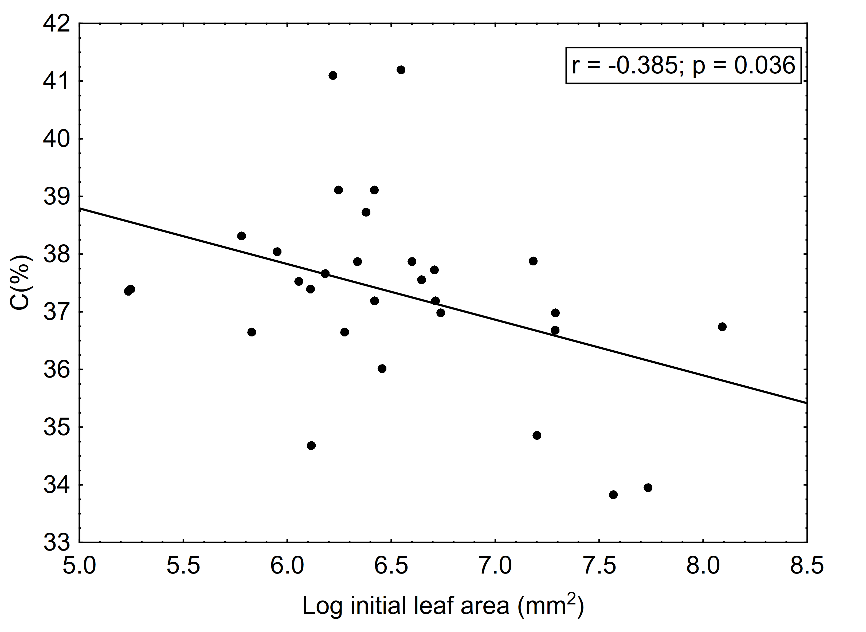


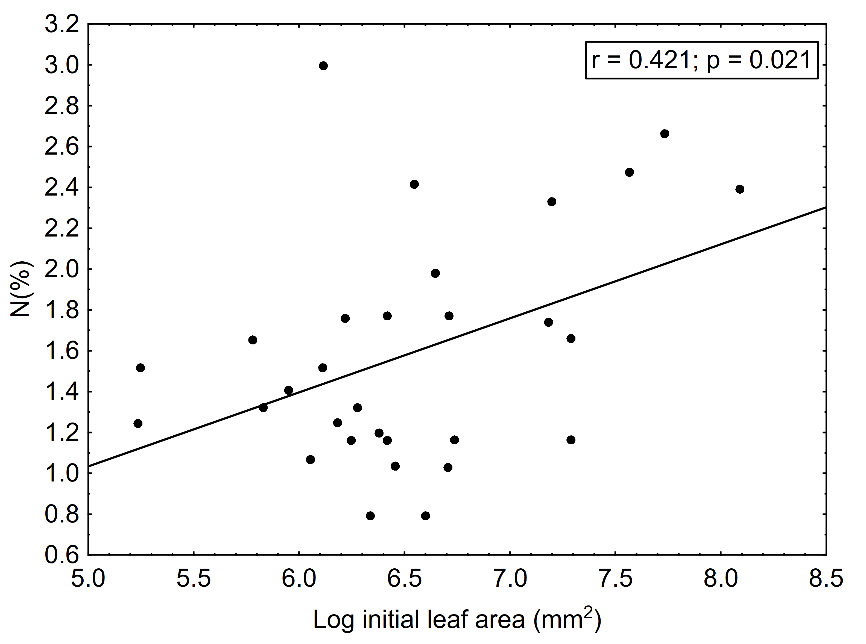


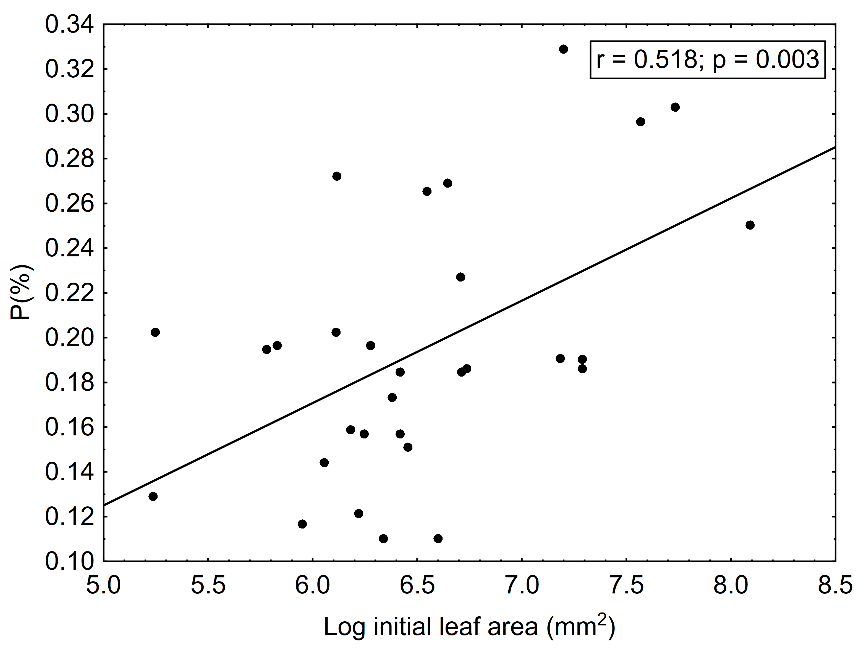

Supplement: Supplementary file 1 — Supplementary file1 (DOCX 1514 kb) [file 41598_2020_67437_MOESM1_ESM.docx]
